# Forensic neuropathology in the past decade: a scoping literature review

**DOI:** 10.1007/s12024-023-00672-9

**Published:** 2023-07-13

**Authors:** Petteri Oura, Antti Hakkarainen, Antti Sajantila

**Affiliations:** 1 Department of Forensic Medicine, Faculty of Medicine, University of Helsinki, P.O. Box 21, Helsinki, FI-00014 Finland; 2 Forensic Medicine Unit, Finnish Institute for Health and Welfare, P.O. Box 30, Helsinki, FI-00271 Finland

**Keywords:** Neuropathology, Literature search, Meta-level attribute, Sample characteristics, Research theme, Methodology, Forensic, Medico-legal

## Abstract

While there has been notable research activity in the field of clinical neuropathology over the recent years, forensic approaches have been less frequent. This scoping literature review explored original research on forensic neuropathology over the past decade (January 1, 2010, until February 12, 2022) using the MEDLINE database. The aims were to (1) analyze the volume of research on the topic, (2) describe meta-level attributes and sample characteristics, and (3) summarize key research themes and methods. Of 5053 initial hits, 2864 fell within the target timeframe, and 122 were included in the review. Only 3–17 articles were published per year globally. Most articles originated from the Europe (39.3%) and Asia (36.1%) and were published in forensic journals (57.4%). A median sample included 57 subjects aged between 16 and 80 years. The most common research theme was traumatic intracranial injury (24.6%), followed by anatomy (12.3%) and substance abuse (11.5%). Key methods included immunotechniques (31.1%) and macroscopic observation (21.3%). Although a number of novel findings were reported, most were of preliminary nature and will require further validation. In order to reach breakthroughs and validate novel tools for routine use, more research input is urged from researchers across the world. It would be necessary to ensure appropriate sample sizes and make use of control groups.

**Supplementary Information:**

The online version contains supplementary material available at 10.1007/s12024-023-00672-9.

## Introduction

Neurological diseases [1] and trauma to the central nervous system (CNS) [2] are common causes of death globally. A comprehensive postmortem examination of the CNS often requires particular expertise and sophisticated tissue processing techniques [3, 4]. Neuropathological expertise is therefore of high value in both clinical [5] and forensic pathology [3]. While there has been notable research activity in the field of clinical neuropathology over the recent years [6–15], forensic approaches appear less frequent [16–20]. However, the role of CNS remains important in the medico-legal practice [21–24], as CNS-related findings may have pivotal significance in cause-of-death investigation [3] and legal proceedings [25, 26].

Literature reviews aid in the efficient utilization of current knowledge. Systematic approaches are needed to summarize and disseminate research findings and identify gaps in the existing literature [27]. However, to the best of the authors’ knowledge, there are no broad-scoped overviews summarizing literature on forensic neuropathology, at least from the past decade. This scoping literature review explored literature on forensic neuropathology from January 1, 2010, until February 12, 2022. The aims were to (1) analyze the volume of research on the topic, (2) describe meta-level attributes and sample characteristics, and (3) summarize key research themes and methods.

## Materials and methods

### Research questions

Scoping reviews are exploited to determine the scope and volume of literature on a given topic and to identify key concepts [28, 29]. In contrast to systematic reviews, scoping reviews are particularly useful when the research question is broad and the body of literature has not been comprehensively reviewed before. We conducted a MEDLINE-based scoping review to explore scientific literature on forensic neuropathology published over the past decade.

The following research questions were formulated in accordance with the general aims of the study:

1. Volume of research
  - What is the volume of original research on forensic neuropathology per year?
2. Meta-level attributes and sample characteristics
  - Which journals publish studies on forensic neuropathology in terms of subspecialty and impact?
  - What is the geographical distribution of publications?
  - What kind of samples are used in terms of size and age distribution?
3. Research themes and methods
  - What are the key concepts, i.e., main research themes and methodological approaches in forensic neuropathology?
  - Are there knowledge gaps?

This review did not aim to summarize or classify particular findings of the studies; however, these are addressed in the supplementary material.

### Search strategy, inclusion, and exclusion criteria

**Fig. 1:**
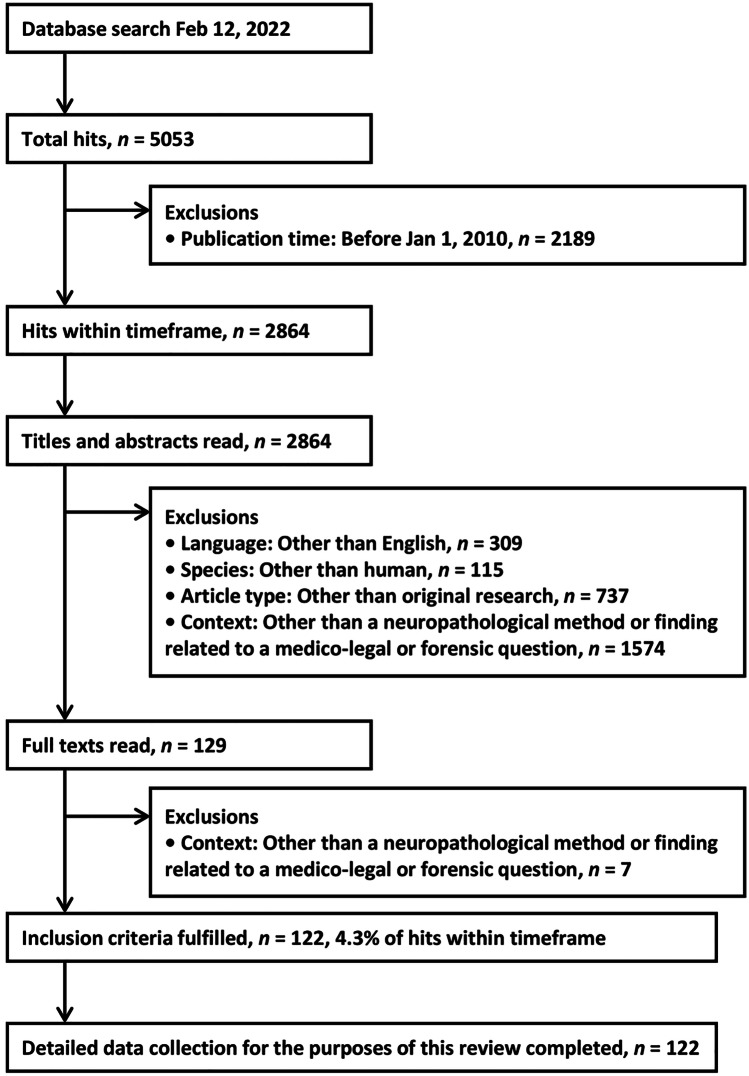
Flowchart demonstrating the article selection process with exclusions

The search strategy was developed by the first author (P.O.) and reviewed by the last author of the paper (A.S.). Table 1 presents the specific search terms used in MEDLINE. Figure 1 is a flowchart demonstrating the article selection process with exclusions.

**Table 1:** Search query in MEDLINE

#	Search term
1	(neuro*[Title/Abstract] OR nervous*[Title/Abstract] OR brain*[Title/Abstract] OR spine*[Title/Abstract] OR spinal*[Title/Abstract] OR cerebr*[Title/Abstract] OR cerebel*[Title/Abstract] OR intracran*[Title/Abstract] OR intracerebr*[Title/Abstract] OR subarachn*[Title/Abstract] OR subdur*[Title/Abstract] OR epidur*[Title/Abstract])
2	(forensic*[Title/Abstract] OR "medico-legal"[Title/Abstract] OR medicolegal[Title/Abstract] OR "medico legal"[Title/Abstract] OR "legal medicine"[Title/Abstract])
3	#1 AND #2

We aimed to find peer-reviewed, original articles that addressed a neuropathological method or finding related to a medico-legal or forensic question in a human sample. A neuropathological method was defined as a macroscopic, microscopic, or other laboratory technique used to examine a tissue sample obtained from the CNS or intracranial structures including vasculature. We focused on English-language articles that were published and indexed in MEDLINE between January 1, 2010, and the database search date. Short communications, retrospective summaries of autopsy findings, and other similar publication types were included if they were original articles based on authentic human samples. Studies that solely focused on postmortem imaging, analysis of body fluids, human identification, or skull fractures without the use of neuropathological methods were excluded.

The search was conducted in the MEDLINE database February 12, 2022. First, P.O. screened all hits on the basis of titles, abstracts, and full texts, if necessary. Each hit was assigned with a rationale for inclusion or exclusion to be later validated by A.S.

### Data extraction and synthesis

Data extraction was performed with the help of an Excel spreadsheet. Table 2 presents the variables collected in the data extraction process. The spreadsheet was a priori planned by P.O. and reviewed by A.S.; an internal pilot was carried out in the beginning of data collection (20 hits from the year 2010). Data extraction was performed on the basis of full texts and potential supplementary material of the articles. While P.O. was primarily responsible for extracting the data, the spreadsheet was reviewed and commented by A.S. A formal risk of bias assessment was not performed, as it is not customary in scoping reviews [28], and was not considered necessary in relation to the present research questions.

**Table 2:** Variables used in the data extraction spreadsheet

Variable	Details
Meta-level attributes	
Name of the first author	-
Year of publication	-
Digital object identifier (DOI)	-
Name of journal	-
Journal area	Categorized as follows: clinical, forensic, pathology, and other
Journal impact factor	Most recent impact factor (2020), collected from Journal Citation Reports, Clarivate Analytics, April 17, 2022
Location	Refers primarily to the country where the study was conducted, and secondarily to the authors’ main affiliation. Initially collected at the country level, then aggregated to the continent level
Sample characteristics	
Sample size	Total sample size, comprising both cases and controls, if applicable
Use of control group	-
Age range	Minimum and maximum ages of the total sample, comprising both cases and controls, if applicable. Further categorized as follows: adults only (refers to subjects aged ≥ 18 years), minors only, both, not stated
Research themes and methods	
Primary theme	Refers to the main research theme that was focused on. Categorized as follows: anatomy, asphyxia and hypoxia, brain edema, brain tissue identification, drowning, hypothermia and hyperthermia, laboratory methods and quality, neurodegeneration, postmortem interval, substance abuse, sudden unexpected death in epilepsy, sudden unexpected death in infancy and childhood, suicide, traumatic intracranial injury, several primary themes, and other
Primary method	Refers to the main method applied in the study. Categorized as follows: conventional measurements (e.g., weight), genetic techniques, histology and conventional staining, immunotechniques, macroscopic observation, several primary methods, and other
Aims and findings	
Aims	Aims of the study. Quoted from the source article
Main findings or conclusions	Quoted from the source article

Data synthesis was conducted in accordance with the predefined research questions. The distributions of publication year, journal characteristics, geographical location, sample characteristics, primary research theme, and methodological approach were tabulated using frequencies with percentages or medians with interquartile ranges, as appropriate. In addition to these summary statistics, a supplementary table containing the extracted data of individual studies was constructed.

## Results

### Literature search

Of 5053 initial hits, 2864 fell within the target timeframe, and 122 were finally included in the review [30–151], corresponding to 4.3% of hits within timeframe (Fig. 1). Most exclusions were due to wrong context (e.g., forensic psychiatry) or article type (i.e., not original article). Individual summaries of the 122 included articles, together with aims and main findings, are presented in Supplementary Table 1.

### Meta-level attributes

Table 3 shows the annual distribution of publications over the review period; 3 to 17 articles were published per year globally. Table 4 is a bibliographic and geographic summary of the studies. Forensic journals were the most common publication channel (57.4%), followed by clinical journals (e.g., general medicine, neurology, or pediatric journals; 23.0%), and pathology journals (8.2%). Median impact factor was 2.3, while 11.5% of studies were published in journals without an Impact Factor. As for geographical distribution, Europe (39.3%) and Asia (36.1%) were the two most common study regions.

**Table 3:** Annual number of included articles over the review period

Year	Number of articles
2010	6
2011	3
2012	14
2013	6
2014	5
2015	8
2016	10
2017	17
2018	15
2019	13
2020	10
2021	11
2022 (until February 12)	4
Total	122

**Table 4:** Bibliographic and geographic summary of the studies (*n* = 122)

	%	*N*
Journal area		
Forensic	57.4	70
Clinical	23.0	28
Pathology	8.2	10
Other	11.5	14
Journal impact		
Impact factor in 2020 (median with IQR)	2.3 (1.6–2.7)	
No impact factor in 2020	11.5	14
Location of study^a^		
Europe	39.3	48
Asia	36.1	44
Americas	18.0	22
Australia and New Zealand	4.9	6
Africa	1.6	2

*IQR* Interquartile range

^a^Location refers primarily to the location where the study was conducted, and secondarily to the authors’ main affiliation

### Sample characteristics

Table 5 is a summary of sample characteristics. A median sample included 57 subjects (interquartile range 29–101; full range 4–1222), which included both cases and controls, if applicable. Control groups were utilized in less than half of the studies (43.4%). Medians of minimum and maximum ages were 16 and 80 years, respectively. A total of 30.3% of studies were based on adult-only samples, another 30.3% had both adults and minors, and 12.3% were based on minors. Subject ages were not stated in over a quarter of the studies (27.0%).

**Table 5:** Sample characteristics of the studies (*n* = 122)

	%	*N*
Sample size^a^		
Number of subjects (median with IQR)	57 (29–101)	
Number of subjects not stated	1.6	2
Control group used	43.4	53
Age of subjects^b^		
Adults only	30.3	37
Minors only	12.3	15
Both	30.3	37
Not stated	27.0	33
Age range^c^		
Minimum age (median with IQR)	16 (0–20)	
Minimum age not stated	26.2	32
Maximum age (median with IQR)	80 (56–91)	
Maximum age not stated	27.0	33

*IQR* interquartile range

^a^Total sample size, comprising both cases and controls, if applicable

^b^Adults refer to subjects aged ≥ 18 years; minors refer to those aged < 18 years

^c^Minimum and maximum ages of the total sample, comprising both cases and controls, if applicable

### Research themes and methods

Lists of research themes and methodological approaches are presented in Table 6. Individual summaries of the articles, together with aims and main findings, are presented in Supplementary Table 1.

**Table 6:** Research themes and methods of the studies (*n* = 122)

	%	*N*
Primary theme^a^		
Traumatic intracranial injury	24.6	30
Anatomy	12.3	15
Substance abuse	11.5	14
Laboratory methods and quality	7.4	9
Sudden unexpected death in infancy and childhood	6.6	8
Neurodegeneration	4.9	6
Suicide	4.1	5
Hypothermia and hyperthermia	3.3	4
Postmortem interval	3.3	4
Asphyxia and hypoxia	2.5	3
Brain edema	2.5	3
Brain tissue identification	2.5	3
Drowning	1.6	2
Sudden unexpected death in epilepsy	1.6	2
Other	9.8	12
Several primary themes	1.6	2
Primary method^b^		
Immunotechniques	31.1	38
Macroscopic observation	21.3	26
Genetic techniques	13.9	17
Conventional measurements (e.g. weight)	9.8	12
Histology and conventional staining	5.7	7
Other	3.3	4
Several primary methods	14.8	18

^a^Primary theme refers to the main research theme that was focused on

^b^Primary method applied in the study

The most common research theme was traumatic intracranial injury (24.6%), which comprised focal and diffuse traumatic brain injuries [30, 31, 39, 43, 54, 55, 64, 83, 85, 86, 94, 106, 107, 115, 116, 120, 123, 130, 132, 139, 150] and traumatic intracranial hemorrhages [30, 47, 54, 57, 58, 65, 83, 87, 99, 113, 114, 130, 141, 150]. Studies often used immunotechniques to identify traumatic changes and estimate the age of injury [39, 85–87, 94, 106, 107, 115, 120, 132, 139]. While some studies described macroscopic injury patterns and combinations in accident and assault scenarios [30, 47, 65, 99, 114, 116, 130, 141], others used conventional histology [57, 58, 113] or several methods [31, 43, 54, 55, 64, 83, 150] to address varying research questions.

The second most common entity was anatomy (12.3%). While most studies described the anatomical variants of vasculature [41, 68, 75, 105, 126, 127, 135] and other intracranial structures [44, 73, 74, 151], some aimed to generate reference values for brain weight in various populations [69, 98, 102, 125]. The main techniques were macroscopic observation, conventional histology, and weight measurement. Moreover, one study evaluated the biomechanical properties of the dura mater [151].

Substance abuse was the primary entity in 11.5% of the studies. Both chronic and acute abuse were represented. The selection of substances included alcohols [38, 52, 82], opioids [38, 100, 109, 117–119], stimulants [38, 53, 81, 136, 147], and other or multiple substances [48, 50, 117, 118]. Immunotechniques [38, 48, 50, 117, 118, 147], genetic techniques [81], or the two together [119, 136] were often utilized to identify brain damage and distinguish substance abuse from other causes of death. Some studies primarily reported macroscopic observations [52, 53, 82, 109] or brain weight [100] among substance abusers.

Laboratory methods were the main focus in 7.4% of the studies [45, 67, 70, 72, 97, 111, 133, 138, 148]. The studies showed notable heterogeneity, addressing technical aspects of, e.g., formalin pigment deposition [45], immunohistochemistry [133], DNA extraction [67], and freezing preparation of putrefied brain tissue [97].

Sudden unexpected deaths in infancy and childhood were addressed in 6.6% of the studies [33, 40, 51, 61, 66, 79, 84, 91]. Immunotechniques [33, 40, 79], genetic techniques [51], conventional histology [91], and brain weight measurement [61] were used to uncover underlying mechanisms and identify brain tissue markers in these cases. Moreover, one study suggested an optimal neuropathologic examination protocol for these deaths in a medico-legal setting [66].

Other research themes were rarely addressed (< 5% each). Neurodegenerative diseases in medico-legal settings were approached using immunotechniques [128, 143, 144], image analysis [129], or a combination of several methods [104, 112]. As for suicide, immunotechniques [32, 89], genetic techniques [62, 88], and brain weight measurement [146] were applied to identify factors that differentiate suicide victims from controls. Brain tissue markers of hypothermia and hyperthermia were studied by means of immunotechniques [78, 140, 145] and genetic techniques [60]. Studies that aimed to improve the estimation of postmortem interval were mainly based on immunotechniques [42, 92] and genetic techniques [134]. Asphyxia and brain hypoxia [35, 77, 108], brain edema [36, 37, 96], brain tissue identification [95, 121, 122], drowning [34, 71], and sudden unexpected death in epilepsy [63, 149] were addressed in two to three individual studies each.

One article studied the markers of traumatic brain injury and mechanical asphyxiation using genetic techniques [49], while another focused on the potential markers of hypothermia, hyperthermia, and intoxication using immunotechniques and genetic techniques [59]. Finally, the following entities had one study each: sudden unexplained nocturnal death syndrome [46], pediatric subdural hemosiderin deposits [56], iron in fetal and infant leptomeninges [80], DNA identification based on brain tissue swab [76], zinc in brain tissue [90], intracranial aneurysms and dissections [101], age estimation [103], electrocution [124], insulin homicide [131], fire fatalities [137], phosphine poisoning [93], and carbon monoxide poisoning [142].

## Discussion

### Main findings

This scoping review identified 122 original articles on forensic neuropathology from the years 2010–2022. Only 3–17 articles were published per year globally. Most articles originated from the Europe and Asia and were published in forensic journals. A median sample included 57 subjects aged between 16 and 80 years. The most common research theme was traumatic intracranial injury, followed by anatomy and substance abuse. Main methods included immunotechniques and macroscopic observation. To the best of the authors’ knowledge, this is the first scoping review to systematically explore literature on forensic neuropathology over the past decade.

### Meta-level considerations

The annual volume of research output was relatively low, which may indicate rather mild research activity in the field globally. It is obvious that breakthroughs will require consistent scientific effort and active involvement of forensic pathologists in research projects. Clinical neuropathology may have outpowered the forensic branch, possibly due to stronger translational potential and active interplay with clinicians [6–15]. Neuropathology is an interdisciplinary field, touching upon neighboring fields such as neurology, neurosurgery, psychiatry, legal medicine, and general pathology. Forensic neuropathology is aligned in the midway between clinical and forensic pathology, often requiring particular expertise from a general forensic pathologist [22]. Interdisciplinary cooperation may thus be the key to increasing research activity in the field.

Articles were widely distributed between journals of various disciplines, which underlines the intersectoral nature of the field. In general, articles were published in international, field-specific journals with a median impact factor of 2.3. However, it is noteworthy that over a tenth of the articles were published in journals with no impact factor whatsoever; anatomic reports appeared to be overrepresented in this subgroup. As for geographical distribution, the vast majority of articles were from European and Asian researchers. Notably, articles from American groups were less common, and only two African articles were published over the entire review period. In order to expedite the development and implementation of forensic neuropathology globally, research input is needed from medico-legal units across the world. Unfortunately, achieving this objective may prove difficult due to resource- and policy-related barriers. It would be important to ensure sufficient personnel resources, methodological expertise, access to research funding, and comprehensible research permit policies for medico-legal data.

### Sample-related considerations

Sample sizes were moderate, with a median of 57 subjects; this included both cases and potential controls. Two articles appeared to lack a clear indication of sample size. Despite the relatively small sample sizes, statistical power calculations were rarely presented. In quantitative studies, power calculations guide sample collection and corroborate the statistical approach [152]. Of note is also the fact that over a half of the studies did not have a control group, which implies that most articles were descriptive in nature. A comparative design is a prerequisite for many scientifically relevant conclusions [153].

Age ranges were generally wide, which increased the generalizability of findings across age groups. However, taking into account the moderate sample sizes, the level of heterogeneity within samples may significantly increase with widening age spans. Over a quarter of studies appeared to lack a clear statement of the minimum and maximum ages of the sample; some reported standard deviations and interquartile ranges instead.

### Research themes, methods, and future directions

Traumatic intracranial injury was the most common research theme. Research activity around the topic is easy to comprehend as traumatic brain injury and intracranial hemorrhages are complex and deadly entities that often present themselves to a forensic pathologist [2, 21]. As neuropathology may have a pivotal role in a cause-of-death investigation [3] or legal proceedings [25, 26], novel tools are needed to identify traumatic changes and estimate the age of injury. However, significant breakthroughs are yet to come.

Alongside traumatic intracranial injury, the top-three research themes included anatomy and substance abuse. Somewhat surprisingly, macroscopic and microscopic anatomy of intracranial structures were among the most popular research themes. Many of these studies reported important findings for neurosurgeons, for example, but appeared to make a minor contribution to the field of forensic neuropathology. Substance abuse, which indeed is a central medico-legal entity [154], was approached from a variety of perspectives method- and substance-wise. However, more research input will be needed to identify substance-specific markers in brain tissue and differentiate substance abuse and intoxication from other causes of death.

Neurodegenerative diseases were addressed in a handful of studies. In spite of the vast research activity in clinical neuropathology, studies in medico-legal samples are also important, as neurodegenerative diseases appear to increase the risk of unnatural deaths [155]. Providing medico-legal units with diagnostic methods that have been validated in medico-legal samples will be of utmost importance. As for sudden unexplained deaths among infants and children, studies have kept chasing potential mechanisms and biomarkers, but again, significant breakthroughs are yet to come.

Although suicide is a major and diverse entity in forensic pathology [156], only a few studies addressed the topic. Considering the obscurity behind predisposing and underlying factors, there should be a lot to achieve mechanism- and prevention-wise. Medico-legal samples may have significant translational potential in this regard. Moreover, only a few studies addressed asphyxia, drowning, hypothermia, hyperthermia, sudden unexpected death in epilepsy, and estimation of postmortem interval. Higher research activity should be directed toward these themes in order to improve postmortem diagnostics.

Immunotechniques, i.e., immunohistochemistry and immunoblotting, were commonly applied to detect potential changes in brain tissue. Genetic techniques were exploited in various approaches such as brain tissue identification and gene expression analysis. A minority of studies used conventional histology as the main method. Although a number of novel findings were reported, most were of preliminary nature and will require further validation. Macroscopic observation of intracranial structures was a common method, but the studies often merely described injury patterns or anatomic variations. The crude measurement of brain weight was also used in some studies, but these often had null findings.

### Limitations of the review

This scoping review had several limitations that should be considered. First, the scope of the literature search was notably broad, and conventional search terms were covered. However, articles that used specific or uncommon terminology may have been omitted. A large number of initial hits were obtained and manually evaluated, which may have reduced the risk of omitting in-scope articles. Second, as the review focused on original articles, emerging research themes may not have been fully covered. Moreover, there is a large body of research that may not be captured in this review even though it is relevant to forensic neuropathologists (e.g., CNS infections and emerging concepts in neurodegenerative diseases). Future reviews are expected to cover these aspects. Finally, as the aim was to explore and summarize original research in the field, there were no particular restrictions on scientific rigor, and no formal bias assessment was performed.

## Conclusion

This scoping literature review explored original research on forensic neuropathology over the years 2010–2022. A total of 122 original articles were eventually included in the synthesis. Traumatic intracranial injury was the most common research theme, immunotechniques being the most commonly applied method. Only 3–17 articles were published per year globally. Although a number of novel findings were reported, most were of preliminary nature and will require further validation. In order to reach breakthroughs and validate novel tools for routine use, more research input is urged in forensic neuropathology from researchers across the world. Interdisciplinary cooperation may be the key to increasing research activity in the field. Researchers should ensure appropriate sample sizes and make use of comparative designs whenever possible.

## Key points

Knowledge of diseases and trauma related to the central nervous system has high value in forensic pathology
This scoping review explored literature on forensic neuropathology from 2010 to 2022
A total of 122 original articles were included, corresponding to 3–17 publications per year globally
4.The most common research theme was traumatic intracranial injury (24.6%), followed by anatomy (12.3%) and substance abuse (11.5%). Key methods included immunotechniques (31.1%) and macroscopic observation (21.3%)
To reach breakthroughs and validate tools for routine practice, more research input is needed from researchers across the world

### Supplementary Information

Below is the link to the electronic supplementary material.

Supplementary file1 (PDF 396 KB)

## Data Availability

This is a review of published literature. The dataset generated and analysed during the study is presented in Supplementary Table 1.

## References

[1] GBD 2016 Neurology Collaborators. Global, regional, and national burden of neurological disorders, 1990–2016: A systematic analysis for the Global Burden of Disease Study 2016. Lancet Neurol. 2019;18:459–80. 10.1016/S1474-4422(18)30499-X PMC6459001 30879893

[2] Rubiano AM, Carney N, Chesnut R, Puyana JC. Global neurotrauma research challenges and opportunities. Nature. 2015;527:S193–7. 10.1038/nature16035 26580327

[3] Kalimo H, Saukko P, Graham D. Neuropathological examination in forensic context. Forensic Sci Int. 2004;146:73–81. 10.1016/j.forsciint.2004.06.022 15542266

[4] Bruner JM, Louis DN, McLendon R, Rosenblum MK, Archambault WT, Most S, et al. The utility of expert diagnosis in surgical neuropathology: Analysis of consultations reviewed at 5 national comprehensive cancer network institutions. J Neuropathol Exp Neurol. 2017;76:189–94. 10.1093/jnen/nlw122 28395084

[5] Iacono D, Geraci-Erck M, Peng H, Bouffard JP. Symmetric bihemispheric postmortem brain cutting to study healthy and pathological brain conditions in humans. J Vis Exp. 2016;118:54602. 10.3791/54602 PMC5226422 28060309

[6] Trejo-Lopez JA, Yachnis AT, Prokop S. Neuropathology of Alzheimer’s disease. Neurotherapeutics. 2022;19:173–85. 10.1007/s13311-021-01146-y PMC9130398 34729690

[7] Kon T, Tomiyama M, Wakabayashi K. Neuropathology of Lewy body disease: Clinicopathological crosstalk between typical and atypical cases. Neuropathology. 2020;40:30–9. 10.1111/neup.12597 31498507

[8] Koga S, Sekiya H, Kondru N, Ross OA, Dickson DW. Neuropathology and molecular diagnosis of synucleinopathies. Mol Neurodegener. 2021;16:83. 10.1186/s13024-021-00501-z PMC8684287 34922583

[9] Lassmann H. The contribution of neuropathology to multiple sclerosis research. Eur J Neurol. 2022;29:2869–2877. 10.1111/ene.15360 PMC9544263 35427431

[10] Clark HB. The neuropathology of autoimmune ataxias. Brain Sci. 2022;12:257. 10.3390/brainsci12020257 PMC8869941 35204019

[11] Fetit R, Hillary RF, Price DJ, Lawrie SM. The neuropathology of autism: A systematic review of post-mortem studies of autism and related disorders. Neurosci Biobehav Rev. 2021;129:35–62. 10.1016/j.neubiorev.2021.07.014 34273379

[12] Cole BL. Neuropathology of pediatric brain tumors: A concise review. Neurosurgery. 2022;90:7–15. 10.1093/neuros/nyab182 34114043

[13] von Spreckelsen N, Kesseler C, Brokinkel B, Goldbrunner R, Perry A, Mawrin C. Molecular neuropathology of brain-invasive meningiomas. Brain Pathol. 2022;32:e13048. 10.1111/bpa.13048 PMC8877755 35213084

[14] Seilhean D. Infections of the central nervous system: Neuropathology. Rev Neurol (Paris). 2019;175:431–5. 10.1016/j.neurol.2019.07.006 31371186

[15] Maiese A, Manetti AC, Bosetti C, del Duca F, la Russa R, Frati P, et al. SARS-CoV-2 and the brain: A review of the current knowledge on neuropathology in COVID-19. Brain Pathol. 2021;31:e13013. 10.1111/bpa.13013 PMC8420197 34390282

[16] Mavroudis I, Kazis D, Chowdhury R, Petridis F, Costa V, Balmus I-M, et al. Post-concussion syndrome and chronic traumatic encephalopathy: Narrative review on the neuropathology, neuroimaging and fluid biomarkers. Diagnostics (Basel). 2022;12:740. 10.3390/diagnostics12030740 PMC8947595 35328293

[17] Zhang L, Lucassen PJ, Salta E, Verhaert PDEM, Swaab DF. Hippocampal neuropathology in suicide: Gaps in our knowledge and opportunities for a breakthrough. Neurosci Biobehav Rev. 2022;132:542–52. 10.1016/j.neubiorev.2021.12.023 34906612

[18] Patodia S, Somani A, Thom M. Review: Neuropathology findings in autonomic brain regions in SUDEP and future research directions. Auton Neurosci. 2021;235:102862. 10.1016/j.autneu.2021.102862 PMC8455454 34411885

[19] McGuone D, Crandall LG, Devinsky O. Sudden unexplained death in childhood: A neuropathology review. Front Neurol. 2020;11:582051. 10.3389/fneur.2020.582051 PMC7596260 33178125

[20] Zwirner J, Kulakofsky R, Fitzek A, Schröder AS, Bohnert S, Franke H, et al. Forensic biomarkers of lethal traumatic brain injury. Int J Legal Med. 2022;136:871–86. 10.1007/s00414-022-02785-2 PMC9005436 35226180

[21] Bertozzi G, Maglietta F, Sessa F, Scoto E, Cipolloni L, di Mizio G, et al. Traumatic brain injury: A forensic approach: A literature review. Curr Neuropharmacol. 2020;18:538–50. 31686630 10.2174/1570159X17666191101123145 PMC7457403

[22] Stewart W, Black M, Kalimo H, Graham DI. Non-traumatic forensic neuropathology. Forensic Sci Int. 2004;146:125–47. 10.1016/j.forsciint.2004.06.025 15542273

[23] Balestrini S, Iacono D, Devinsky O, Mcguone D, Crandall LG. Sudden unexplained death in childhood: A neuropathology review. Front Neurol. 2020;11:582051. 10.3389/fneur.2020.582051 PMC7596260 33178125

[24] MacKenzie JM. Examining the decomposed brain. Am J Forensic Med Pathol. 2014;35:265–70. 10.1097/PAF.0000000000000111 25384305

[25] Kresak JL, Zehe S, Reichard RR. What every neuropathologist needs to know: Neuropathology and the US legal system. J Neuropathol Exp Neurol. 2019;78:291–3. 10.1093/jnen/nly131 30753558

[26] Whitwell H, Milroy C, du Plessis D. Forensic Neuropathology. 2nd Ed. London: CRC Press; 2021.

[27] Pham MT, Rajić A, Greig JD, Sargeant JM, Papadopoulos A, Mcewen SA. A scoping review of scoping reviews: Advancing the approach and enhancing the consistency. Res Synth Methods. 2014;5:371–85. 10.1002/jrsm.1123 PMC4491356 26052958

[28] Munn Z, Peters MDJ, Stern C, Tufanaru C, McArthur A, Aromataris E. Systematic review or scoping review? Guidance for authors when choosing between a systematic or scoping review approach. BMC Med Res Methodol. 2018;18:143. 10.1186/s12874-018-0611-x PMC6245623 30453902

[29] Peters MDJ, Godfrey CM, Khalil H, McInerney P, Parker D, Soares CB. Guidance for conducting systematic scoping reviews. Int J Evid Based Healthc. 2015;13:141–6. 10.1097/XEB.0000000000000050 26134548

[30] Aghakhani K, Heidari M, Ameri M, Mehrpisheh S, Memarian A. Characteristics of traumatic brain injury among accident and falling down cases. Acta Med Iran. 2015;53:652–5. 26615380

[31] Al-Sarraj S, Fegan-Earl A, Ugbade A, Bodi I, Chapman R, Poole S, et al. Focal traumatic brain stem injury is a rare type of head injury resulting from assault: A forensic neuropathological study. J Forensic Leg Med. 2012;19:144–51. 22391000 10.1016/j.jflm.2011.12.015

[32] Alvarado-Esquivel C, Mendoza-Larios LA, García-Dolores F, Sánchez-Anguiano LF, Antuna-Salcido EI, Hernández-Tinoco J, et al. Association between Toxoplasma gondii infection in brain and a history of depression in suicide decedents: A cross-sectional study. Pathogens. 2021;10:1313. 10.3390/pathogens10101313 PMC8539687 34684262

[33] Ambrose N, Waters KA, Rodriguez ML, Bailey K, Machaalani R. Neuronal apoptosis in the brainstem medulla of sudden unexpected death in infancy (SUDI), and the importance of standardized SUDI classification. Forensic Sci Med Pathol. 2018;14:42–56. 29460253 10.1007/s12024-018-9954-1

[34] An J-L, Ishida Y, Kimura A, Kondo T. Immunohistochemical examination of intracerebral aquaporin-4 expression and its application for differential diagnosis between freshwater and saltwater drowning. Int J Legal Med. 2011;125:59–65. 21069372 10.1007/s00414-010-0523-8

[35] Bartschat S, Fieguth A, Könemann J, Schmidt A, Bode-Jänisch S. Indicators for acute hypoxia-An immunohistochemical investigation in cerebellar Purkinje-cells. Forensic Sci Int. 2012;223:165–70. 22980140 10.1016/j.forsciint.2012.08.023

[36] Bauer M, Deigendesch N, Wittig H, Scheurer E, Lenz C. Tissue sample analysis for post mortem determination of brain edema. Forensic Sci Int. 2021;323:110808. 10.1016/j.forsciint.2021.110808 33971505

[37] Bauer M, Gerlach K, Scheurer E, Lenz C. Analysis of different post mortem assessment methods for cerebral edema. Forensic Sci Int. 2020;308:110164. 10.1016/j.forsciint.2020.110164 32014814

[38] Bohnert S, Georgiades Kosmas, ·, Monoranu C-M, Bohnert · Michael, Büttner A, Ondruschka B. Quantitative evidence of suppressed TMEM119 microglial immunohistochemistry in fatal morphine intoxications. Int J Legal Med. 2021;135:2315–22. 34553260 10.1007/s00414-021-02699-5 PMC8523458

[39] Bohnert S, Seiffert A, Trella S, Bohnert M, Distel L, Ondruschka B, et al. TMEM119 as a specific marker of microglia reaction in traumatic brain injury in postmortem examination. Int J Legal Med. 2020;134:2167–76. 32719959 10.1007/s00414-020-02384-z PMC7578160

[40] Bright FM, Byard RW, Vink R, Paterson DS. Medullary serotonin neuron abnormalities in an Australian cohort of sudden infant death syndrome. J Neuropathol Exp Neurol. 2017;76:864–73. 10.1093/jnen/nlx071 28922849

[41] Bruno-Mascarenhas MA, Ramesh VG, Venkatraman S, Mahendran J v., Sundaram S. Microsurgical anatomy of the superior sagittal sinus and draining veins. Neurol India. 2017;65:794–800. 10.4103/neuroindia.NI_644_16 28681754

[42] Campell ZK, Kwon I, Finley SJ, Lee Y, Javan GT. Talin: A potential protein biomarker in postmortem investigations. J Forensic Leg Med. 2016;44:188–91. 10.1016/j.jflm.2016.10.020 27825046

[43] Castellani RJ, Smith M, Bailey K, Perry G, Dejong JL. Neuropathology in consecutive forensic consultation cases with a history of remote traumatic brain injury. J Alzheimers Dis. 2019;72:683–91. 10.3233/JAD-190782 31609691

[44] Cavdar S, Solmaz B, Tanis Ö, Guler O, Dalcik H, Aydogmus E, et al. Anatomic variations of the human falx cerebelli and its association with occipital venous sinuses. Br J Neurosurg. 2021;35:306–12. 32781846 10.1080/02688697.2020.1793907

[45] Chatzopoulos K, Treeck B van, Venable E, Serla V, Wirth T, Amirahmadi F, et al. Formalin pigment artifact deposition in autopsy tissue: predisposing factors, patterns of distribution and methods for removal. Forensic Sci Med Pathol. 2020;16:435–41. 10.1007/s12024-020-00240-5 32201924

[46] Chen Z, Mu J, Chen X, Dong H. Sudden unexplained nocturnal death syndrome in Central China (Hubei): A 16-year retrospective study of autopsy cases. Medicine (Baltimore). 2016;95:1–6. 10.1097/MD.0000000000002882 PMC4782858 26945374

[47] Cheshire EC, Biggs MJP, Hollingbury FE, Fitzpatrick-Swallow VL, Prickett TRA, Malcomson RDG. Frequency of macroscopic intradural hemorrhage with and without subdural hemorrhage in early childhood autopsies. Forensic Sci Med Pathol. 2019;15:184–90. 30915608 10.1007/s12024-019-00103-8 PMC6505489

[48] Chindemi C, Cirielli V, Cima L, Danzi O, Raniero D, Tagliaro F, et al. Autophagy pathways in drug abusers after forensic autopsy: LC3B, ph-mTOR and p70S6K analysis. Med Sci Law. 2019;59:49–56. 30852985 10.1177/0025802419828910

[49] Chung U, Seo J-S, Kim Y-H, Hoon Son G, Hwang J-J. Quantitative analyses of postmortem heat shock protein mRNA profiles in the occipital lobes of human cerebral cortices: Implications in cause of death. Mol Cells. 2012;34:473–80. 10.1007/s10059-012-0214-z PMC3887795 23135635

[50] Cirielli V, Cima L, Chindemi C, Danzi O, Ghimenton C, Eccher A, et al. Cortical expression of the polysialylated isoform of the neural cell adhesion molecule on brain tissue to recognize drug-related death. Am J Forensic Med Pathol. 2018;39:8–13. 10.1097/PAF.0000000000000366 29293100

[51] Danusso R, Alfonsi G, Ferrero S, Lavezzi AM, Lattuada D. Mitochondrial DNA content: A new potential biomarker for sudden infant death syndrome. Pediatr Res. 2022;92:1282–1287. 10.1038/s41390-021-01901-z 35102299

[52] Darke S, Duflou J, Forensic Pathologist C, Torok M, Officer R, Prolov T, et al. Toxicology, circumstances and pathology of deaths from acute alcohol toxicity. J Forensic Leg Med. 2013;20:1122–5. 24237834 10.1016/j.jflm.2013.09.002

[53] Darke S, Lappin J, Kaye S, Duflou J. Clinical characteristics of fatal methamphetamine-related stroke: A national study. J Forensic Sci. 2018;63:735–9. 10.1111/1556-4029.13620 28833107

[54] Davceva N, Janevska V, Ilievski B, Petrushevska G, Popeska Z. The occurrence of acute subdural haematoma and diffuse axonal injury as two typical acceleration injuries. J Forensic Leg Med. 2012;19:480–4. 23084313 10.1016/j.jflm.2012.04.022

[55] Davceva N, Janevska V, Ilievski B, Spasevska J, Jovanovic R. The importance of the detail forensic-neuropathological examination in the determination of the diffuse brain injuries. Soud Lek. 2012;57:2–6. 22724588

[56] del Bigio MR, Phillips SM. Retroocular and subdural hemorrhage or hemosiderin deposits in pediatric autopsies. J Neuropathol Exp Neurol. 2017;76:313–22. 10.1093/jnen/nlx010 28340081

[57] Delteil C, Kolopp M, Capuani C, Humez S, Boucekine M, Leonetti G, et al. Histological dating of subarachnoid hemorrhage and retinal hemorrhage in infants. Forensic Sci Int. 2019;303:109952. 10.1016/j.forsciint.2019.109952 31546166

[58] Delteil C, Humez S, Boucekine M, Jouvet A, Hedouin V, Fanton L, et al. Histological dating of subdural hematoma in infants. Int J Legal Med. 2019;133:539–46. 10.1007/s00414-018-1980-8 30554266

[59] Du SH, Tan XH, Zhao R, Zhao D, Xue Y, Wang HJ, et al. Molecular pathology of cerebral TNF-α, IL-1β, iNOS and Nrf2 in forensic autopsy cases with special regard to deaths due to environmental hazards and intoxication. Forensic Sci Med Pathol. 2017;13:409–16. 10.1007/s12024-017-9896-z 28776218

[60] Du Y, Xu J-T, Jin H-N, Zhao R, Zhao D, Du S-H, et al. Increased cerebral expressions of MMPs, CLDN5, OCLN, ZO1 and AQPs are associated with brain edema following fatal heat stroke. Sci Rep. 2017;7:1691. 10.1038/s41598-017-01923-w PMC5431794 28490769

[61] Elliott JA, Vink R, Jensen L, Byard RW. Brain weight-body weight ratio in sudden infant death syndrome revisited. Med Sci Law. 2012;52:207–9. 22619376 10.1258/msl.2012.011136

[62] Erbay L, Karhdag R, Oruc M, Cigremis Y, Celbis O. Association of BDNF/TRKB and NGF/TRKA levels in postmortem brain with major depression and suicide. Psychiatr Danub. 2021;33:491–8. 34928896 10.24869/psyd.2021.491

[63] Esen Melez İ, Arslan M, Melez D, Şanli AN, Koç S. Sudden unexpected death in epilepsy: A retrospective autopsy study of 112 epileptic patients. Arch Neuropsychiatry. 2017;54:225–33. 10.5152/npa.2016.14863 PMC5630100 29033634

[64] Florou C, Zorilă A, Zorilă M, Marinescu M, Andrei C, Păvăloiu R, et al. Clinico-statistical and morphological aspects of severe traumatic brain injuries. Rom J Morphol Embryol. 2016;57:391–400. 27516010

[65] Flugt A, Frost L, Søndergaard C, Milidou I. Lethal abusive head trauma in infancy in Denmark from 2000 to 2011. Dan Med J. 2021;68:AO8200604. 33829989

[66] Folkerth RD, Nunez J, Georgievskaya Z, McGuone D. Neuropathologic examination in sudden unexpected deaths in infancy and childhood: Recommendations for highest diagnostic yield and cost-effectiveness in forensic settings. Acad Forensic Pathol. 2017;7:182–99. 31239973 10.23907/2017.020 PMC6474536

[67] Funabashi KS, Barcelos D, Visoná I, e Silva SM, Almeida ML, e Sousa PO, et al. DNA extraction and molecular analysis of non-tumoral liver, spleen, and brain from autopsy samples: The effect of formalin fixation and paraffin embedding. Pathol Res Pract. 2012;208:584–91. 22920941 10.1016/j.prp.2012.07.001

[68] García Corredor N, Forero Porras P, Ballesteros Acuña L. Morphological evaluation of the distal medial striated artery. A study with cadaverous material. Colomb Med (Cali). 2020;51:e204440. 10.25100/cm.v51i3.4440 PMC7744106 33402753

[69] Gholamzadeh S, Zarenezhad M, Montazeri M, Zareikordshooli M, Sadeghi G, Malekpour A, et al. Statistical analysis of organ morphometric parameters and weights in South Iranian adult autopsies. Medicine (Baltimore). 2017;96:e6447. 10.1097/MD.0000000000006447 PMC5457842 28538362

[70] Gielda L, Rigg S. Extraction of amplifiable DNA from embalmed human cadaver tissue. BMC Res Notes. 2017;10:737. 10.1186/s13104-017-3066-y PMC5729266 29237482

[71] Girela-López E, Beltran-Aroca CM, Dye A, Gill JR. Epidemiology and autopsy findings of 500 drowning deaths. Forensic Sci Int. 2022;330:111137. 10.1016/j.forsciint.2021.111137 34894613

[72] Hanson E, Ballantyne J. Human organ tissue identification by targeted RNA deep sequencing to aid the investigation of traumatic injury. Genes (Basel). 2017;8:319. 10.3390/genes8110319 PMC5704232 29125589

[73] Haque MA, Khalil M, Khalil M, Sultana SZ, Mannan S, Rahman M, et al. Morphometry of Purkinje cell body of cerebellum in Bangladeshi cadaver. Mymensingh Med J Bangladesh. 2010;19:504–9. 20956889

[74] Haque MA, Khalil M, Sultana SZ, Mannan S, Uddin MM, Hossain M, et al. Morphometric study of dentate nucleus of cerebellum in Bangladeshi cadaver. Mymensingh Med J Bangladesh. 2015;24:25–33. 25725664

[75] Hashemi R, Mahmoodi R, Amirjamshidi A. Variations in the anatomy of the Willis’ circle: A 3-year cross-sectional study from Iran (2006–2009). Are the distributions of variations of circle of Willis different in different populations? Result of an anatomical study and review of literature. Surg Neurol Int. 2013;4:65. 10.4103/2152-7806.112185 PMC3680999 23772335

[76] Helm K, Matzenauer C, Neuhuber F, Monticelli F, Meyer H, Pittner S, et al. Suitability of specific soft tissue swabs for the forensic identification of highly decomposed bodies. Int J Legal Med. 2021;135:1319–27. 33880634 10.1007/s00414-021-02601-3 PMC8205910

[77] Hu Y, Tian L, Ma K, Han L, Li W, Hu L, et al. ER stress-related protein, CHOP, may serve as a biomarker of mechanical asphyxia: a primary study. Int J Legal Med. 2022;136:1091–104. 35122137 10.1007/s00414-021-02770-1

[78] Ishikawa T, Yoshida C, Michiue T, Große Perdekamp M, Pollak S, Maeda H. Immunohistochemistry of catecholamines in the hypothalamic-pituitary-adrenal system with special regard to fatal hypothermia and hyperthermia. Leg Med (Tokyo). 2010;12:121–7. 20207184 10.1016/j.legalmed.2010.01.004

[79] Jack E, Haas E, Haddix TL. Evaluation of the presence and distribution of leptomeningeal inflammation in SIDS/SUDI cases and comparison with a hospital-based cohort. Childs Nerv Syst. 2019;35:2391–7. 10.1007/s00381-019-04268-z 31270575

[80] Jack E, Fennelly NK, Haddix T. The inflammatory cellular constituents of foetal and infant leptomeninges: A survey of hospital-based autopsies without trauma. Childs Nerv Syst. 2014;30:911–7. 24402186 10.1007/s00381-013-2348-5 PMC3983874

[81] Johnson MM, David JA, Michelhaugh SK, Schmidt CJ, Bannon MJ. Increased heat shock protein 70 gene expression in the brains of cocaine-related fatalities may be reflective of postdrug survival and intervention rather than excited delirium. J Forensic Sci. 2012;57:1519–23. 22803793 10.1111/j.1556-4029.2012.02212.x PMC4530606

[82] Karayel F, Turan A, Sav A, Pakis I, Akyildiz E, Ersoy G. Methanol intoxication. Am J Forensic Med Pathol. 2010;31:34–6. 10.1097/PAF.0b013e3181c160d9 20010293

[83] Kibayashi K, Shimada R, Nakao KI, Ro A. Analysis of pituitary lesions in fatal closed head injury. Am J Forensic Med Pathol. 2012;33:206–10. 21030847 10.1097/PAF.0b013e3181fe33e8

[84] Kinney HC, Cryan JB, Haynes RL, Paterson DS, Haas EA, Othon, et al. Dentate gyrus abnormalities in sudden unexplained death in infants: Morphological marker of underlying brain vulnerability. Acta Neuropathol. 2015;129:65–80. 25421424 10.1007/s00401-014-1357-0 PMC4282685

[85] Kobek M, Jankowski Z, Szala J, Gąszczyk-Ozarowski Z, Pałasz A, Skowronek R. Time-related morphometric studies of neurofilaments in brain contusions. Folia Neuropathol. 2016;54:50–8. 10.5114/fn.2016.58915 27179221

[86] Krohn M, Drebler J, Bauer M, Schober K, Franke H, Ondruschka B. Immunohistochemical investigation of S100 and NSE in cases of traumatic brain injury and its application for survival time determination. J Neurotrauma. 2015;32:430–40. 25211554 10.1089/neu.2014.3524

[87] Krywanczyk A, Bundock EA. Quantifying macrophages and hemosiderin in pediatric dura mater. J Forensic Sci. 2018;63:902–5. 28851096 10.1111/1556-4029.13634

[88] Krzyzanowska M, Steiner J, Karnecki K, Kaliszan M, Brisch R, Wiergowski M, et al. Decreased ribosomal DNA transcription in dorsal raphe nucleus neurons differentiates between suicidal and non-suicidal death. Eur Arch Psychiatry Clin Neurosci. 2016;266:217–24. 10.1007/s00406-015-0655-4 PMC4819736 26590846

[89] Kurtulus Dereli A, Demırci GN, Dodurga Y, Özbal S, Cankurt U, Boz B, et al. Evaluation of human pineal gland acetylserotonin O-methyltransferase immunoreactivity in suicide: A preliminary study. Med Sci Law. 2018;58:233–8. 10.1177/0025802418797178 30185109

[90] Lech T, Sadlik J. Zinc in postmortem body tissues and fluids. Biol Trance Elem Res. 2011;142:11–7. 10.1007/s12011-010-8747-5 20549399

[91] Leitner DF, McGuone D, William C, Faustin A, Askenazi M, Snuderl M, et al. Blinded review of hippocampal neuropathology in sudden unexplained death in childhood reveals inconsistent observations and similarities to explained paediatric deaths. Neuropathol Appl Neurobiol. 2022;e12746. 10.1111/nan.12746 PMC8777468 34164845

[92] Lesnikova I, Schreckenbach MN, Kristensen MP, Papanikolaou LL, Hamilton-Dutoit S. Usability of immunohistochemistry in forensic samples with varying decomposition. Am J Forensic Med Pathol. 2018;39:185–91. 10.1097/PAF.0000000000000408 29794805

[93] Liang Y, Tong F, Huang F, Liu Y, Zhu L, le Grange JM, et al. Pathological changes induced by phosphine poisoning: A study on 8 children. Int J Legal Med. 2020;134:217–28. 10.1007/s00414-019-02169-z 31713064

[94] Lier J, Ondruschka B, Bechmann I, Dreßler J. Fast microglial activation after severe traumatic brain injuries. Int J Legal Med. 2020;134:2187–93. 32372233 10.1007/s00414-020-02308-x PMC7578125

[95] Lindenbergh A, van den Berge M, Oostra R-J, Cleypool C, Bruggink A, Kloosterman A, et al. Development of a mRNA profiling multiplex for the inference of organ tissues. Int J Legal Med. 2013;127:891–900. 23839651 10.1007/s00414-013-0895-7

[96] Lundesgaard Eidahl JM, Opdal SH, Rognum TO, Stray-Pedersen A. Postmortem evaluation of brain edema: An attempt with measurements of water content and brain-weight-to-inner-skull-circumference ratio. J Forensic Leg Med. 2019;64:1–6. 10.1016/j.jflm.2019.03.003 30877967

[97] Matoba K, Hyodoh H, Murakami M, Matoba T, Saito A, Feng F, et al. Freezing preparation for macroscopic forensic investigation in putrefied brain. Leg Med (Tokyo). 2017;26:6–10. 10.1016/j.legalmed.2017.01.005 28549549

[98] Mehrpour O, Sheikhazadi A, Hasan Ghadyani M, Hooshyar H. Brain weight of Iranian population; The first report. J Forensic Leg Med. 2010;17:426–31. 21056877 10.1016/j.jflm.2010.08.012

[99] Mohd Saman SA, Jothee S, Nor FM, Shafie MS. The pattern of injuries among motorcyclists in fatal road traffic accidents: An autopsy-based study. Am J Forensic Med Pathol. 2021;42:141–6. 10.1097/PAF.0000000000000639 33346978

[100] Molina D, Vance K, Coleman M, Hargrove V. Testing an age-old adage: Can autopsy findings be of assistance in differentiating opioid versus cardiac deaths? J Forensic Sci. 2020;65:112–6. 31483504 10.1111/1556-4029.14174

[101] Mori S, Takahashi S, Hayakawa A, Saito K, Takada A, Fukunaga T. Fatal intracranial aneurysms and dissections causing subarachnoid hemorrhage: An epidemiological and pathological analysis of 607 legal autopsy cases. J Stroke Cerebrovasc Dis. 2018;27:486–93. 29108808 10.1016/j.jstrokecerebrovasdis.2017.09.031

[102] Mubbunu L, Bowa K, Petrenko V, Silitongo M. Correlation of internal organ weights with body weight and body height in normal adult Zambians: A case study of Ndola Teaching Hospital. Anat Res Int. 2018;2018:4687538. 29850249 10.1155/2018/4687538 PMC5932513

[103] Naue J, Sänger T, Hoefsloot HCJ, Lutz-Bonengel S, Kloosterman AD, Verschure PJ. Proof of concept study of age-dependent DNA methylation markers across different tissues by massive parallel sequencing. Forensic Sci Int Genet. 2018;36:152–9. 10.1016/j.fsigen.2018.07.007 30031222

[104] Nishida N, Yoshida K, Hata Y, Arai Y, Kinoshita K. Pathological features of preclinical or early clinical stages of corticobasal degeneration: a comparison with advanced cases. Neuropathol Appl Neurobiol. 2015;41:893–905. 25708668 10.1111/nan.12229

[105] Nyasa C, Mwakikunga A, Tembo LH, Dzamalala C, Ihunwo AO. Anatomical variations and morphometric properties of the circulus arteriosus cerebri in a cadaveric Malawian population. Folia Morphol (Warsz). 2021;80:820–6. 10.5603/FM.a2020.0142 33330970

[106] Oerter S, Förster C, Bohnert M. Validation of sodium/glucose cotransporter proteins in human brain as a potential marker for temporal narrowing of the trauma formation. Int J Legal Med. 2019;133:1107–14. 30073510 10.1007/s00414-018-1893-6

[107] Olczak M, Poniatowski Ł, Kwiatkowska M, Samojłowicz D, Tarka S, Wierzba-Bobrowicz T. Immunolocalization of dynein, dynactin, and kinesin in the cerebral tissue as a possible supplemental diagnostic tool for traumatic brain injury in postmortem examination. Folia Neuropathol. 2019;57:51–62. 31038188 10.5114/fn.2019.83831

[108] Olczak M, Chutorański D, Kwiatkowska M, Samojłowicz D, Tarka S, Wierzba-Bobrowicz T. Bystin (BYSL) as a possible marker of severe hypoxic-ischemic changes in neuropathological examination of forensic cases. Forensic Sci Med Pathol. 2018;14:26–30. 29349722 10.1007/s12024-017-9942-x PMC5830468

[109] Pelletier DE, Andrew TA. Common findings and predictive measures of opioid overdoses. Acad Forensic Pathol. 2017;7:91–8. 31239961 10.23907/2017.011 PMC6474474

[110] Pelletti G, Garagnani M, Barone R, Boscolo-Berto R, Rossi F, Morotti A, et al. Validation and preliminary application of a GC–MS method for the determination of putrescine and cadaverine in the human brain: a promising technique for PMI estimation. Forensic Sci Int. 2019;297:221–7. 10.1016/j.forsciint.2019.01.025 30831414

[111] Preusse-Prange A, Modrow J-H, Schwark T, von Wurmb-Schwark N. Detection of constitutive and inducible HSP70 proteins in formalin fixed human brain tissue. Forensic Sci Int. 2014;235:62–7. 10.1016/j.forsciint.2013.12.004 24447452

[112] Priemer DS, Folkerth RD. Dementia in the forensic setting: Diagnoses obtained using a condensed protocol at the Office of Chief Medical Examiner, New York City. J Neuropathol Exp Neurol. 2021;80:724–30. 10.1093/jnen/nlab059 34388235

[113] Rao M, Singh D, Vashista R, Sharma S. Dating of acute and subacute subdural haemorrhage: A histo-pathological study. J Clin Diagn Res. 2016;10:HC01–7. 10.7860/JCDR/2016/19783.8141 PMC5020299 27630864

[114] Rebollo-Soria MC, Arregui-Dalmases C, Sánchez-Molina D, Velázquez-Ameijide J, Galtés I. Injury pattern in lethal motorbikes-pedestrian collisions, in the area of Barcelona, Spain. J Forensic Leg Med. 2016;43:80–4. 10.1016/j.jflm.2016.07.009 27494040

[115] Romero Tirado M, Pampin JMB, Gómez RG. Dating of traumatic brain injury in forensic cases using immunohistochemical markers (I): Neurofilaments and β-amyloid precursor protein. Am J Forensic Med Pathol. 2018;39:201–7. 10.1097/PAF.0000000000000412 29901458

[116] Rungruangsak K, Poriswanish N. Pathology of fatal diffuse brain injury in severe non-penetrating head trauma. J Forensic Leg Med. 2021;82:102226. 10.1016/j.jflm.2021.102226 34375839

[117] Sadat-Shirazi MS, Soltani H, Nikpour N, Haghshenas M, Khalifeh S, Mokri A, et al. Alteration of orexin-A and PKCα in the postmortem brain of pure-opioid and multi-drug abusers. Neuropeptides. 2020;83:102074. 10.1016/j.npep.2020.102074 32741526

[118] Sadat-Shirazi M-S, Zarrindast M-R, Ashabi G. Oxidative stress enzymes are changed in opioid abusers and multidrug abusers. J Clin Neurosci. 2020;72:365–8. 31926663 10.1016/j.jocn.2019.12.064

[119] Sadat-Shirazi MS, Zarrindast MR, Daneshparvar H, Ziaie A, Fekri M, Abbasnezhad E, et al. Alteration of dopamine receptors subtypes in the brain of opioid abusers: A postmortem study in Iran. Neurosci Lett. 2018;687:169–76. 10.1016/j.neulet.2018.09.043 30268777

[120] Sakai K, Fukuda T, Iwadate K. Immunohistochemical analysis of the ubiquitin proteasome system and autophagy lysosome system induced after traumatic intracranial injury: Association with time between the injury and death. Am J Forensic Med Pathol. 2014;35:38–44. 24317096 10.1097/PAF.0000000000000067

[121] Samsuwan J, Muangsub T, Yanatatsaneejit P, Mutirangura A, Kitkumthorn N. Combined bisulfite restriction analysis for brain tissue identification. Forensic Sci Int. 2018;286:42–5. 10.1016/j.forsciint.2018.02.032 29558685

[122] Sauer E, Extra A, Caché P, Courts C. Identification of organ tissue types and skin from forensic samples by microRNA expression analysis. Forensic Sci Int Genet. 2017;28:99–110. 28193507 10.1016/j.fsigen.2017.02.002

[123] Schober K, Ondruschka B, Dreßler J, Abend M. Detection of hypoxia markers in the cerebellum after a traumatic frontal cortex injury: A human postmortem gene expression analysis. Int J Legal Med. 2015;129:701–7. 10.1007/s00414-014-1129-3 PMC4475240 25432860

[124] Shaha KK, Joe AE. Electrocution-related mortality: A retrospective review of 118 deaths in Coimbatore, India, between January 2002 and December 2006. Med Sci Law. 2010;50:72–4. 20593598 10.1258/msl.2010.010008

[125] Sheikhazadi A, Shahabeddin Sadr S, Hasan Ghadyani M, Kazem Taheri S, Asghar Manouchehri A, Nazparvar B, et al. Study of the normal internal organ weights in Tehran’s population. J Forensic Leg Med. 2010;17:78–83. 20129426 10.1016/j.jflm.2009.07.012

[126] Siddiqi H, Tahir M, Lone K. Variations in cerebral arterial circle of Willis in adult Pakistani population. J Coll Physicians Surg Pak. 2013;23:615–9. 24034183

[127] Sultana N, Khalil M, Khan MK, Kabir A, Farjan S, Ismatsara M, et al. Variation in the position and diameter of basilar artery in different ages of Bangladeshi people. Mymensingh Med J. 2018;27:504–7. 30141438

[128] Takayama M, Kashiwagi M, Matsusue A, Waters B, Hara K, Ikematsu N, et al. Quantification of immunohistochemical findings of neurofibrillary tangles and senile plaques for a diagnosis of dementia in forensic autopsy cases. Leg Med (Tokyo). 2016;22:82–9. 10.1016/j.legalmed.2016.08.007 27591545

[129] Takayama M, Kashiwagi M, Matsusue A, Waters B, Hara K, Ikematsu N, et al. Quantification of neuropathological findings by image data for the diagnosis of dementia in forensic autopsy cases. J Med Invest. 2016;63:114–8. 27040064 10.2152/jmi.63.114

[130] Tolescu S, Zorila M, Serbanescu M, Kamal K, Zorila G, Dumitru I, et al. Severe traumatic brain injury (TBI)-A seven-year comparative study in a Department of Forensic Medicine. Rom J Morphol Embryol. 2020;61:95–103. 10.47162/RJME.61.1.10 PMC7728107 32747899

[131] Tong F, Wu R, Huang W, Yang Y, Zhang L, Zhang B, et al. Forensic aspects of homicides by insulin overdose. Forensic Sci Int. 2017;278:9–15. 28686962 10.1016/j.forsciint.2017.06.015

[132] Trautz F, Franke H, Bohnert S, Hammer N, Müller W, Stassart R, et al. Survival-time dependent increase in neuronal IL-6 and astroglial GFAP expression in fatally injured human brain tissue. Sci Rep. 2019;9:11771. 31417126 10.1038/s41598-019-48145-w PMC6695416

[133] Trautz F, Dreßler J, Stassart R, Müller W, Ondruschka B. Proposals for best-quality immunohistochemical staining of paraffin-embedded brain tissue slides in forensics. Int J Legal Med. 2018;132:1103–9. 10.1007/s00414-017-1767-3 29299666

[134] van den Berge M, Wiskerke D, Gerretsen R, Tabak J, Sijen T. DNA and RNA profiling of excavated human remains with varying postmortem intervals. Int J Legal Med. 2016;130:1471–80. 27627902 10.1007/s00414-016-1438-9

[135] Vasović L, Trandafilović M, Jovanović I, Ugrenović S, Vlajković S. Vertebral and/or basilar dolichoectasia in human adult cadavers. Acta Neurochir. 2012;154:1477–88. 10.1007/s00701-012-1400-7 22664729

[136] Wang Q, Ishikawa T, Michiue T, Zhu B-L, Guan D-W, Maeda H. Molecular pathology of brain matrix metalloproteases, claudin5, and aquaporins in forensic autopsy cases with special regard to methamphetamine intoxication. Int J Legal Med. 2014;128:469–74. 24522335 10.1007/s00414-014-0972-6

[137] Wang Q, Ishikawa T, Michiue T, Zhu B-L, Guan D-W, Maeda H. Molecular pathology of brain edema after severe burns in forensic autopsy cases with special regard to the importance of reference gene selection. Int J Legal Med. 2013;127:881–9. 23702882 10.1007/s00414-013-0868-x

[138] Wang Q, Ishikawa T, Michiue T, Zhu B-L, Guan D-W, Maeda H. Stability of endogenous reference genes in postmortem human brains for normalization of quantitative real-time PCR data: Comprehensive evaluation using geNorm, NormFinder, and BestKeeper. Int J Legal Med. 2012;126:943–52. 10.1007/s00414-012-0774-7 23010907

[139] Wang Q, Ishikawa T, Michiue T, Zhu B-L, Guan D-W, Maeda H. Quantitative immunohistochemical analysis of human brain basic fibroblast growth factor, glial fibrillary acidic protein and single-stranded DNA expressions following traumatic brain injury. Forensic Sci Int. 2012;221:142–51. 10.1016/j.forsciint.2012.04.025 22607979

[140] Wang Q, Ishikawa T, Michiue T, Zhu B-L, Guan D-W, Maeda H. Evaluation of human brain damage in fatalities due to extreme environmental temperature by quantification of basic fibroblast growth factor (bFGF), glial fibrillary acidic protein (GFAP), S100b and single-stranded DNA (ssDNA) immunoreactivities. Forensic Sci Int. 2012;219:259–64. 22285501 10.1016/j.forsciint.2012.01.015

[141] Wong B, Ong BB, Milne N. The source of haemorrhage in traumatic basal subarachnoid haemorrhage. J Forensic Leg Med. 2015;29:18–23. 10.1016/j.jflm.2014.09.012 25572079

[142] Yarid NA, Harruff RC. Globus pallidus necrosis unrelated to carbon monoxide poisoning: Retrospective analysis of 27 cases of basal ganglia necrosis. J Forensic Sci. 2015;60:1484–7. 10.1111/1556-4029.12838 26258901

[143] Yoshida K, Hata Y, Ichimata S, Nishida N. Tau and amyloid-β pathology in Japanese forensic autopsy series under 40 years of age: Prevalence and association with APOE genotype and suicide risk. J Alzheimers Dis. 2019;72:641–52. 10.3233/JAD-190196 31594218

[144] Yoshida K, Hata Y, Kinoshita K, Takashima S, Tanaka K, Nishida N. Incipient progressive supranuclear palsy is more common than expected and may comprise clinicopathological subtypes: A forensic autopsy series. Acta Neuropathol. 2017;133:809–23. 28064358 10.1007/s00401-016-1665-7

[145] Yoshida C, Ishikawa T, Michiue T, Quan L, Maeda H. Postmortem biochemistry and immunohistochemistry of chromogranin A as a stress marker with special regard to fatal hypothermia and hyperthermia. Int J Legal Med. 2011;125:11–20. 19760428 10.1007/s00414-009-0374-3

[146] Zedler B, Flaig B, Ackermann H, Parzeller M, Bratzke H. Brain weight in completed suicide and other cases of death-comparison of recent and previous studies. Int J Legal Med. 2014;128:295–301. 10.1007/s00414-013-0913-9 24048502

[147] Zhang Z, Gong Q, Feng X, Zhang D, Quan L. Astrocytic clasmatodendrosis in the cerebral cortex of methamphetamine abusers. Forensic Sci Res. 2017;2:139–44. 30483632 10.1080/20961790.2017.1280890 PMC6197099

[148] Zhang H, Zhang P, Ma K, Lv Y, Li W, Luo C, et al. The selection of endogenous genes in human postmortem tissues. Sci Justice. 2013;53:115–20. 23601718 10.1016/j.scijus.2012.11.005

[149] Zhuo L, Zhang Y, Zielke HR, Levine B, Zhang X, Chang L, et al. Sudden unexpected death in epilepsy: Evaluation of forensic autopsy cases. Forensic Sci Int. 2012;223:171–5. 22999232 10.1016/j.forsciint.2012.08.024

[150] Zorilă A, Zorilă M, Marinaş M, Ţolescu R, Zorilă G, Florou C, et al. Evaluation of brain injuries in children deceased due to head trauma. Rom J Morphol Embryol. 2017;58:1417–28. 29556636

[151] Zwirner J, Scholze M, Neil Waddell J, Ondruschka B, Hammer N. Mechanical properties of human dura mater in tension-An analysis at an age range of 2 to 94 years. Sci Rep. 2019;9:16655. 31723169 10.1038/s41598-019-52836-9 PMC6853942

[152] Wade A. Fear or favour? Statistics in pathology. J Clin Pathol. 2000;53:16–8. 10.1136/jcp.53.1.16 PMC1731052 10767848

[153] Grimes DA, Schulz KF. Compared to what? Finding controls for case-control studies. Lancet. 2005;365:1429–33. 10.1016/S0140-6736(05)66379-9 15836892

[154] Büttner A. Review: The neuropathology of drug abuse. Neuropathol Appl Neurobiol. 2011;37:118–34. 10.1111/j.1365-2990.2010.01131.x 20946118

[155] An JH, Lee KE, Jeon HJ, Son SJ, Kim SY, Hong JP. Risk of suicide and accidental deaths among elderly patients with cognitive impairment. Alzheimers Res Ther. 2019;11:32. 10.1186/s13195-019-0488-x PMC6460725 30975186

[156] Byard RW, Austin A. The role of forensic pathology in suicide. Forensic Sci Med Pathol. 2010;7:1–2. 10.1007/s12024-010-9186-5 20645136

