# Supplementary material for: Forensic neuropathology in the past decade: a scoping literature review
Source: Forensic Sci Med Pathol. 2023 Jul 13;20(2):724–35. doi: 10.1007/s12024-023-00672-9 (PMC11297074; doi:10.1007/s12024-023-00672-9)
Supplement: Supplementary file 1 — Supplementary file1 (PDF 396 KB) [file 12024_2023_672_MOESM1_ESM.pdf]

Supplementary Table 1. Full list of included articles, listed by first author's last name and publication year.

First author	Year	DOI	Journal	Location[1]	Primary theme[2]	Primary method[3]	N[4]	Age[5] (years)		Aims (quoted[6])	Main findings/conclusions (quoted[6])
								Min	Max		
Aghakhani	2015	N/a	Acta Med Iran	Iran	Traumatic intracranial injury	Macroscopic observation	237	2	80	"To compare the characteristics of traumatic brain injury among accident and falling down cases."	"From accident group, subdural hemorrhage in 84.7%, subarachnoid hemorrhage in 88%, epidural hemorrhage in 22.4%, [and] contusion in 76.5% of cases. In falling down group subdural hemorrhage in 79.6%, subarachnoid hemorrhage in 87%, epidural hemorrhage in 18.5%, [and] contusion in 61.1% of cases. There was no significant difference between these two groups."
Al-Sarraj	2012	10.1016/ j.jflm.2011.12.015	J Forensic Leg Med	UK	Traumatic intracranial injury	Several primary methods	12	20	80	"To establish the neuropathological criteria of the focal traumatic brainstem injury and correlate them with clinical history and the mechanism of injury."	"Focal traumatic brainstem injury occurs most likely due to direct impact at the back of the head or stretching forces affecting the brainstem in cases of complex fall from height and after assault, particularly those associated with kicks. It is a commonly fatal brain damage, which needed to be differentiated from other causes of brainstem haemorrhages."
Alvarado-Esquivel	2021	10.3390/ pathogens10101313	Pathogens	Mexico	Suicide	Immunotechniques	87	10	90	"[To assess] the association between Toxoplasma gondii infection of the central nervous system and suicide in a sample of decedents who committed suicide in Mexico City."	"The prevalence of Toxoplasma gondii infection in brain in suicide victims was 8.0%. Toxoplasma gondii infection in brain is associated with a history of depression in suicide decedents."
Ambrose	2018	10.1007/ s12024-018-9954-1	Forensic Sci Med Pathol	Australia	Sudden unexpected death in infancy and childhood	Immunotechniques	68	0	1	"In addition to using active caspase-3 and Terminal deoxynucleotidyl transferase mediated deoxyuridine triphosphate nick-end labelling, we include active caspase-9, a specific marker of the intrinsic pathway, to elucidate involvement of this particular apoptotic pathway in a newly characterized sudden unexpected death in infancy cohort."	"The expression of apoptotic markers was similar in explained sudden unexpected death in infancy and sudden infant death syndrome I infants. However, Terminal deoxynucleotidyl transferase mediated deoxyuridine triphosphate nick-end labelling expression was greater in the cuneate, vestibular and hypoglossal nuclei and active caspase-3 expression was lower in the arcuate nucleus in sudden infant death syndrome II compared to explained sudden unexpected death in infancy. Compared to sudden infant death syndrome I infants, sudden infant death syndrome II infants had greater Terminal deoxynucleotidyl transferase mediated deoxyuridine triphosphate nick-end labelling expression in the dorsal motor nucleus of the vagus and greater active caspase-9 expression in the medial and spinal vestibular nuclei. Changes in apoptotic expression predominated in sudden infant death syndrome II infants."
An	2011	10.1007/ s00414-010-0523-8	Int J Legal Med	Japan	Drowning	Immunotechniques	70	0	90	"[To immunohistochemically examine] aquaporin 1 and aquaporin 4 expression in human brain and its suitability for postmortem differentiation between freshwater and saltwater drowning."	"Immunohistochemical analysis of intracerebral aquaporin 4 expression would be forensically useful for differentiation between freshwater and saltwater drowning."
Bartschat	2012	10.1016/ j.forsciint.2012.08.023	Forensic Sci Int	Germany	Asphyxia and hypoxia	Immunotechniques	141	3	98	"To examine the effect of hypoxia on the immunohistochemically detected distribution of calcium-binding protein-D28k, hypoxia-inducible factor-1α and its downstream factor vascular endothelial growth factor in the cerebellar Purkinje-cells, and to consider whether these can be used to support a diagnosis of acute hypoxia."	"Hypoxia-inducible factor-1α and vascular endothelial growth factor were not suitable indicators, whereas detection of a decreasing concentration of calcium-binding protein-D28k supports a diagnosis of acute hypoxia."
Bauer	2020	10.1016/ j.forsciint.2020.110164	Forensic Sci Int	Switzerland	Brain edema	Conventional measurements (e.g. weight)	31	20	86	"To validate the formula of Radojevic et al. [for determining the presence or absence of cerebral edema using the intracranial dimensions and the cerebral weight, against] the macroscopic signs rated at autopsy." (Analysis phase 1)	"[There was a poor agreement] with the macroscopic edema evaluation." (Analysis phase 1)
Bauer	2021	10.1016/ j.forsciint.2021.110808	Forensic Sci Int	Switzerland	Brain edema	Several primary methods	34	18	N/a	"To compare the histology, wet-dry weight and normalized cerebral weight method and to validate their results with the current gold standard to assess brain edema."	"Both the histological and the wet-dry weight method show limited benefits for the classification of brain edema and the histology analysis is highly observer dependent. The normalized cerebral weight method, however, reveals a significant effect between the edematous and nonedematous cases when classifying according to the gold standard."
Bohnert	2020	10.1007/ s00414-020-02384-z	Int J Legal Med	Germany	Traumatic intracranial injury	Immunotechniques	48	5	95	"To investigate TMEM119 for the first time as a useful microglia-specific marker in forensic assessments of traumatic causes of death, e.g., traumatic brain injury, together with a detailed insight into the phagocytic function and the neuroinflammation capacity of monocytes."	"We validate a specific and robustly expressed as well as fast reacting microglia marker, TMEM119, which distinguishes microglia from resident and infiltrating macrophages and thus offers a great potential for the estimation of the minimum survival time after traumatic brain injury."
Bohnert	2021	10.1007/ s00414-021-02699-5	Int J Legal Med	Germany	Substance abuse	Immunotechniques	36	22	85	"To investigate the potential of TMEM119 as a useful microglia-specific marker in a "forensic preselection" of fatalities with single substance abuse (morphine, ethanol, methamphetamine) and to evaluate microglial contribution to brain pathology in respect to its cellular diversity in combination with the phagocytic function and the infiltrating capacity of monocytes."	"We could document the lowest density of TMEM119-positive cells in morphine deaths with highly significant differences to the control densities in all three regions investigated. In ethanol and methamphetamine deaths, the expression of TMEM119 was comparable to cell densities in controls. The results indicate that the immunoreaction in brain tissue is different in these groups depending on the drug type used for abuse."
Bright	2017	10.1093/ jnen/nlx071	J Neuropathol Exp Neurol	Australia	Sudden unexpected death in infancy and childhood	Immunotechniques	69	0	1	"To determine whether previously reported abnormalities in the expression and morphology of 5-hydroxytryptamine neurons within the medullary 5-hydroxytryptamine network in sudden infant death syndrome exist in an independent sudden infant death syndrome cohort from South Australia."	"Compared with controls sudden infant death syndrome cases had significantly higher [medullary] 5-hydroxytryptamine neuron numbers and density in addition to significantly altered 5-hydroxytryptamine neuron morphology. "
Bruno-Mascarenhas	2017	10.4103/ neuroindia.NI_644_16	Neurol India	India	Anatomy	Macroscopic observation	60	20	59	"[To describe] the normal variations in the disposition of the superior sagittal sinus and the number and direction of the draining veins in the Indian population."	"The position of the superior sagittal sinus was variable and was up to within 1 cm on either side of the sagittal suture. The origin of the superior sagittal sinus varied from the level of foramen caecum to a little posterior from the foramen caecum."
Campell	2016	10.1016/ j.jflm.2016.10.020	J Forensic Leg Med	US	Postmortem interval	Immunotechniques	18	19	72	"To determine if there is a correlation between protein expression in cadaver tissues and postmortem interval."	"Talin protein levels steadily decreased with increasing postmortem interval. Talin protein levels were statistically significant between postmortem intervals of 24 versus 48 h and 24 versus 72 h."
Castellani	2019	10.3233/ JAD-190782	J Alzheimers Dis	US	Traumatic intracranial injury	Several primary methods	30	21	92	"[To] examine neuropathological findings in consecutive decedents with a history of traumatic brain injury who presented for forensic autopsy and were subsequently referred for neuropathological consultation."	"Cerebral contusion and post-traumatic epilepsy may be over-represented in civilian traumatic brain injury, while structural brain damage from trauma is the predominant cause of morbidity following traumatic brain injury. We found no evidence that traumatic brain injury initiates a progressive proteinopathy."
Cavdar	2021	10.1080/ 02688697.2020.1793907	Br J Neurosurg	Turkey	Anatomy	Macroscopic observation	49	18	89	"To define the variations and the morphology of the falx cerebelli and its relations with the occipital sinus in human cases."	"Among the 49 falx cerebelli 73.5% were classified as normal. The dural-venous variation in the posterior cranial fossa can be problematic in various diagnostic and operative procedures of this region."
Chatzopoulos	2020	10.1007/ s12024-020-00240-5	Forensic Sci Med Pathol	US	Laboratory methods and quality	Histology and conventional staining	31	N/a	N/a	"To identify types of deaths and types of tissue that are most likely to be affected by formalin pigment deposition. A secondary aim was to assess the utility of two relatively simple laboratory methods for the removal of formalin pigment."	"The organs organs least affected [by formalin deposition] were brain and lung. Formalin pigment deposition correlated with length of postmortem interval. Histologic patterns of formalin deposition included the endothelial lining of vessels [and] perinuclear compartment of neurons. The alcoholic ammoniumhydroxide method was slightly more effective than the alkylphenol ethoxylate method for removing formalin pigment, though both methods were effective."
Chen	2016	10.1097/ MD.0000000000002882	Medicine (Baltimore)	China	Other	Conventional measurements (e.g. weight)	98	16	55	"To assess the epidemiology characteristics of sudden unexplained nocturnal death syndrome in central China and observe its underlying pathological changes."	"Compared to sudden noncardiac deaths, the weights of brain, heart, and lungs had no statistical difference in sudden unexplained nocturnal death syndrome."
Cheshire	2019	10.1007/ s12024-019-00103-8	Forensic Sci Med Pathol	UK	Traumatic intracranial injury	Macroscopic observation	27	0	2	"To describe the occurrence of macroscopically visible intra-falcine and intra-tentorial hemorrhages, observed by two consultant forensic pathologists, in a series of photographs from neonatal, infant and early childhood post-mortem examinations."	"Subdural hemorrhage was recorded for 8 out of 27 cases. Of these 8 cases, it was agreed that 4 had intradural hemorrhage. We report a much lower frequency of macroscopic intradural hemorrhage occurring alongside subdural hemorrhage than previous studies."
Chindemi	2019	10.1177/ 0025802419828910	Med Sci Law	Italy	Substance abuse	Immunotechniques	35	19	65	"To investigate the signalling pathways of autophagy in brain tissues from drug abusers."	"Autophagy pathways were activated in our series, and 56% of drug abusers showed simultaneous LC3B–p70S6K immunoexpression on tissue from the parietal cortex and cerebellum."
Chung	2012	10.1007/ s10059-012-0214-z	Mol Cells	Korea	Several primary entities	Genetic techniques	56	25	69	"To quantitatively analyze a subgroup of brain-enriched heat shock protein messenger RNA transcripts particularly in the post-mortem human occipital lobes, thus gaining useful clues on their differential profiles by the cause of death, such as traumatic injury, mechanical asphyxiation, or sudden cardiac death."	"Heat shock protein A2 messenger RNA levels were higher in subjects who died due to mechanical asphyxiation, compared with those who died by traumatic injury. By contrast, heat shock protein A7 and A13 gene transcripts were much higher in the traumatic injury group than in the asphyxiation and sudden cardiac death groups."
Cirielli	2018	10.1097/ PAF.0000000000000366	Am J Forensic Med Pathol	Italy	Substance abuse	Immunotechniques	35	21	55	"To investigate the polysialylated isoform of the neural cell adhesion molecule immunoexpression on cortex tissues as an indicator of brain damage in a cohort of drug-related deaths."	"The polysialylated isoform of the neural cell adhesion molecule immunoexpression in the neuronal soma and dendritic spines was observed in 72% of drug abusers and in 20% of control subjects. Drug abusers were statistically more positive for the polysialylated isoform of the neural cell adhesion molecule than control subjects. The expression of polysialylated isoform of the neural cell adhesion molecule in the parietal cortex could be an indicator of brain damage due to drug abuse."
Danusso	2022	10.1038/ s41390-021-01901-z	Pediatr Res	Italy	Sudden unexpected death in infancy and childhood	Genetic techniques	42	N/a	N/a	"To determine whether these mitochondrial alterations [in mitochondrial DNA levels] can serve as biomarkers for both sudden infant and early childhood deaths."	"[There was] higher mitochondrial DNA content in the cerebral cortex of the sudden infant death syndrome cases than the controls."
Darke	2013	10.1016/ j.jflm.2013.09.002	J Forensic Leg Med	Australia	Substance abuse	Macroscopic observation	83	22	83	"To determine the levels of systemic disease of fatal cases of acute alcohol toxicity." (Aim #3)	"The only alcohol-related organic brain syndrome pathology identified at autopsy was vermal cerebellar degeneration."

First author	Year	DOI	Journal	Location[1]	Primary theme[2]	Primary method[3]	N[4]	Age[5] (years)		Aims (quoted[6])	Main findings/conclusions (quoted[6])
								Min	Max		
Darke	2018	10.1111/ 1556-4029.13620	J Forensic Sci	Australia	Substance abuse	Macroscopic observation	38	23	57	"To determine the clinical characteristics of a national case series of fatal methamphetamine-related stroke that occurred in Australia over the period 2009–2015."	"The stroke was hemorrhagic in 37 of 38 cases. In 21.1% of cases, the stroke was purely parenchymal and, in 18.4%, involved purely the subarachnoid space. A ruptured berry aneurysm was present in 31.6% and in 68.8% of initial subarachnoid hemorrhages. There was evidence of systemic hypertension in 8 of 25 cases in which full autopsy findings were available."
Davceva	2012	10.1016/ j.jflm.2012.04.022	J Forensic Leg Med	Macedonia	Traumatic intracranial injury	Several primary methods	80	5	94	"To study the occurrence of acute subdural haematoma and diffuse axonal injury in three different types of traumatic events: traffic accidents, falls and assaults (blow)."	"Acute subdural haematoma is more likely to occur in cases of simple fall, assaults and cyclists and diffuse axonal injury is more typical for vehicular traffic accidents and cases of falling from a considerable height."
Davceva	2012	N/a	Soud Lek	Macedonia	Traumatic intracranial injury	Several primary methods	80	N/a	N/a	"To emphasize the necessity of the forensic-neuropathological examination in the determination of the diffuse brain injuries."	"The occurrence of the diffuse brain injuries in the absence of any other massive intracranial lesion has been established in 17.7% of the cases. Hence, forensic-neuropathological examination has been the only way to establish the diagnosis of the brain injury that caused a serious brain failure and in most of them occurred as a concrete cause of death."
Del Bigio	2017	10.1093/ jnen/nlx010	J Neuropathol Exp Neurol	Canada	Other	Histology and conventional staining	62	0	2	"To document the presence of blood or hemosiderin in the retroocular orbital tissues as well as in the cranial and spinal subdural compartments of infants and young children."	"In 53 cases of non-traumatic death, approximately 70% had blood or hemosiderin within the orbital fat, ocular muscles, and parasagittal cranial and/or cervical spinal subdural compartment. None had evidence of hemorrhage within the optic nerve sheath. Premature birth was less likely associated with orbital tissue hemorrhage. Caesarean section birth (mainly nonelective) was not associated with lower prevalence. Residual hemosiderin was identifiable up to 36 weeks postnatal age. Cardiopulmonary resuscitation (performed in the majority of cases) was not associated with acute hemorrhage. In 9 traumatic deaths, 6 had blood and/or hemosiderin within the optic nerve sheath."
Delteil	2019	10.1016/ j.forsciint.2019.109952	Forensic Sci Int	France	Traumatic intracranial injury	Histology and conventional staining	83	0	2	"To propose a dating system for subarachnoid hemorrhage and retinal hemorrhage in the infant in order to improve the precision and reliability of forensic histopathological expert reports on abusive head trauma."	"For subarachnoid hemorrhage, histopathological changes were significantly correlated with posttraumatic interval for the appearance of red blood cells, of fibrino-plaquetted organization, the quantity of lymphocytes and macrophages and the presence or absence of siderophages, collagen and fibroblast formation and presence or absence of neovascularization. For retinal hemorrhage, histopathological changes were significantly correlated with posttraumatic interval for the appearance of red blood cells, the presence or absence of siderophages and sclerosis of the retina. Our dating system improves the precision and reliability of forensic pathological expert examination of abusive head trauma, when subdural hematoma are not available, for age estimation in infants. The study of retinal hemorrhage histomorphological changes does not allow for reliable dating."
Delteil	2019	10.1007/ s00414-018-1980-8	Int J Legal Med	France	Traumatic intracranial injury	Histology and conventional staining	73	0	2	"To propose a dating system for subdural hematoma in the infant in order to improve the precision and reliability of forensic histopathological expert reports on non-accidental head injury."	"Histopathological changes were significantly correlated with posttraumatic interval for the appearance of red blood cells and the presence or absence of siderophages, and regarding the dura mater, the quantity of lymphocytes, macrophages, and siderophages; presence or absence of hematoidin deposits; collagen and fibroblast formation; neomembrane thickness; and presence or absence of neovascularization. Dating systems for subdural hematoma in adults are not applicable to infants. Notably, neomembrane of organized connective tissue is formed earlier in infants than in adults. Our dating system improves the precision and reliability of forensic pathological expert examination of non-accidental head injury, particularly for age estimation of subdural hematoma in infants."
Du	2017	10.1038/ s41598-017-01923-w	Sci Rep	China	Hypothermia and hyperthermia	Several primary methods	46	N/a	N/a	"[To analyze] the gene expressions of matrix metalloproteinase 2, matrix metalloproteinase 9, claudin 5, occludin, zona occludens protein 1, aquaporin 1 and aquaporin 4, in the brains of forensic autopsy cases, using reverse transcription quantitative polymerase chain reaction, combined with immunohistochemical detections, to investigate the molecular pathology of brain edema in fatal heat stroke cases with special regard to the importance of reference gene selection."	"[There were] increased calibrated normalized relative quantity values of matrix metalloproteinase 9, claudin 5, occludin, zona occludens protein 1 and aquaporin 4 in heat stroke cases. In immunostaining, only aquaporin 4 showed more intense staining in most heat stroke cases."
Du	2017	10.1007/ s12024-017-9896-z	Forensic Sci Med Pathol	China	Several primary entities	Several primary methods	200	13	83	"[To analyze] the gene expressions of interleukin-1β, tumor necrosis factor-α, inducible nitric oxide synthase and nuclear factor erythroid-2-related factor-2, using reverse transcription quantitative polymerase chain reaction, combined with immunohistochemical detections, to investigate the molecular pathology in the brains in forensic autopsy cases with special regard to deaths due to environmental hazards and intoxication."	"[There was] higher expression of interleukin-1β, tumor necrosis factor-α and inducible nitric oxide synthase, and lower expression of nuclear factor erythroid-2-related factor-2 in methamphetamine intoxication and hyperthermia cases, higher expression of inducible nitric oxide synthase in phenobarbital intoxication cases, and higher expression of nuclear factor erythroid-2-related factor-2 in phenobarbital intoxication and hypothermia cases."
Elliott	2012	10.1258/ msl.2012.011136	Med Sci Law	Australia	Sudden unexpected death in infancy and childhood	Conventional measurements (e.g. weight)	67	0	0	"To determine whether the brain–body weight ratio is increased in sudden infant death syndrome."	"Although there was a trend towards higher brain–body weight ratios in sudden infant death syndrome, this did not reach significance. The role of brain weight in the aetiology of sudden infant death syndrome remains controversial."
Erbay	2021	10.24869/ psyd.2021.491	Psychiatr Danub	Turkey	Suicide	Genetic techniques	86	18	80	"To compare the expression profiles of brain-derived neurotrophic factor, tyrosine kinase B, nerve growth factor and tyrosine kinase A in postmortem brain tissue of patients who committed suicide due to depression with those who did not die from suicide and did not have a psychiatric history."	"Brain-derived neurotrophic factor, nerve growth factor, tyrosine kinase A and tyrosine kinase B values were found to be lower in the suicide group compared to the control group."
Esen Melez	2017	10.5152/ npa.2016.14863	Arch Neuropsychiatry	Turkey	Sudden unexpected death in epilepsy	Macroscopic observation	112	0	87	"To present autopsy findings of patients who had a prior diagnosis of epilepsy."	"The cause of death was determined to be sudden unexpected death in epilepsy in 35.7% [of cases who had a prior diagnosis of epilepsy]. A possible macroscopic and/or microscopic epileptic focus was present in 20.5% [of the sudden unexpected death in epilepsy cases]."
Florou	2016	N/a	Rom J Morphol Embryol	Romania	Traumatic intracranial injury	Several primary methods	622	N/a	N/a	"[To assess] the clinico-statistical and morphological aspects of severe traumatic brain injury, based on the autopsy reports from the Institute of Forensic Medicine, Craiova, Romania."	"The forensic examination highlighted the severity of cerebral meningeal lesions, the most frequent being cerebral and vascular lesions. The histopathological and immunohistochemical examinations emphasized various microscopic changes in accordance with the severity of the trauma and the time passed from impact until death."
Flugt	2021	N/a	Dan Med J	Denmark	Traumatic intracranial injury	Macroscopic observation	8	0	0	"To estimate the incidence of lethal abusive head trauma in infancy in Denmark from 2000 through 2011 and to describe autopsy findings and information from police files for lethal abusive head trauma cases."	"We identified eight cases of lethal infant abusive head trauma (incidence: 1.04 per 100,000 person years). They all had recent subdural and/or subarachnoid haemorrhage. We also found a high prevalence of retinal haemorrhage and fractures."
Folkerth	2017	10.23907/ 2017.020	Acad Forensic Pathol	US	Sudden unexpected death in infancy and childhood	Several primary methods	53	0	4	"To determine the optimal examination protocol that would be both high-yield for important diagnostic details and also resource-conscious, given the constraints under which many forensic agencies must operate."	"The sections most valuable for detection of relevant pathology, and thus recommended for routine sampling, were: 1) bilateral hippocampus; 2) cerebral cortex and leptomeninges; and 3) pons or medulla."
Funabashi	2012	10.1016/ j.prp.2012.07.001	Pathol Res Pract	Brazil	Laboratory methods and quality	Genetic techniques	40	N/a	N/a	"To compare the three DNA extration methods most used, either from non-tumor specimens of liver, spleen, and brain obtained by autopsy 8–24 h post-mortem, which were fixed in formalin and embedded in paraffin or frozen."	"The best results of amplification of the B-actin gene were observed in recent samples, showing a small variation between tissues, especially in brain samples extracted with phenol–chloroform."
Garcia Corredor	2020	10.25100/ cm.v51i3.4440	Colomb Med (Cali)	Colombia	Anatomy	Macroscopic observation	71	18	65	"To evaluate the morphology of the distal medial striated artery."	"The distal medial striated artery was presented in 1.4% and 4.2% duplicated in the right and left hemisphere respectively. Agenesis was presented in 2.8% in the left hemisphere. The main qualitative finding was the sinuous trajectory that was observed in 57.7% in right side and 45.1% in the left hemisphere."
Gholamzadeh	2017	10.1097/ MD.0000000000006447	Medicine (Baltimore)	Iran	Anatomy	Conventional measurements (e.g. weight)	501	15	98	"To determine the normal range of organ morphometric parameters and weights in south Iranian populations."	"[Brain was] significantly heavier in males compared to females. Moreover, brain became heavier as one got older."
Gielda	2017	10.1186/ s13104-017-3066-y	BMC Res Notes	US	Laboratory methods and quality	Genetic techniques	4	N/a	N/a	"[To develop] a simple and low-cost method of DNA isolation utilizing brain tissue from embalmed cadaver tissue."	"We report the development of a simple, reliable, and low-cost method of DNA isolation utilizing brain tissue from embalmed tissues that could be used for polymerase chain reaction amplification and genetic analysis."
Girela-López	2022	10.1016/ j.forsciint.2021.111137	Forensic Sci Int	US	Drowning	Conventional measurements (e.g. weight)	500	N/a	N/a	"To examine the weights of the brains in drowning deaths."	"Weights of the brains are higher in salt water [than fresh water drownings], although [they are] mostly dependent on other variables such as body mass index and decomposition."
Hanson	2017	10.3390/ genes8110319	Genes (Basel)	US	Laboratory methods and quality	Genetic techniques	5	N/a	N/a	"To develop molecular methods that forensic geneticists can use to identify internal organ tissue using massively parallel sequencing methodology."	"We have developed a prototype massively parallel sequencing messenger RNA profiling assay for organ tissue identification that is designed to definitively identify 10 organ/tissue types using a targeted panel of 46 messenger RNA biomarkers. The identifiable organs and tissues include brain."
Haque	2010	N/a	Mymensingh Med J	Bangladesh	Anatomy	Histology and conventional staining	30	0	60	"To make a standard [of the vertical and transverse diameter of the cerebellar Purkinje cell bodies] for Bangladeshi population."	"Significant differences were observed in the diameter of Purkinje cell body in different age groups but no significant differences was observed between sexes."
Haque	2015	N/a	Mymensingh Med J	Bangladesh	Anatomy	Conventional measurements (e.g. weight)	63	0	60	"To make a standard [of the length and breadth of the cerebellar dentate nucleus] for Bangladeshi population."	"The mean (± SD) length and breadth of dentate nucleus was 8.619 ± 2.995 mm and 14.770 ± 3.604 mm respectively."
Hashemi	2013	10.4103/ 2152-7806.112185	Surg Neurol Int	Iran	Anatomy	Macroscopic observation	200	16	71	"To find the variations of the anatomy of the vessels in the circle of Willis in Iranian people."	"Of the specimens examined, 34.5% were compatible with the typical anatomy of the circle of Willis. Hypoplasia of the posterior communicating arteries was the most common variation in our study."

First author	Year	DOI	Journal	Location[1]	Primary theme[2]	Primary method[3]	N[4]	Age[5] (years)		Aims (quoted[6])	Main findings/conclusions (quoted[6])
								Min	Max		
Helm	2021	10.1007/ s00414-021-02601-3	Int J Legal Med	Austria	Other	Genetic techniques	28	N/a	N/a	"To establish whether a swab of a certain type of soft tissue is still suitable for a valid forensic identification even at advanced degrees of putrefaction."	"We found variable degrees of suitability of different types of soft tissue swabs for DNA-based identification. Swabs of the aortic wall, the urinary bladder wall and brain tissue yielded the best results — in descending order — even at advanced levels of decay."
Hu	2022	10.1007/ s00414-021-02770-1	Int J Legal Med	China	Asphyxia and hypoxia	Immunotechniques	64	15	88	"[To examine] the expression level of C/EBP homologous protein and glucose-regulated protein 78 in brain tissue of samples died of different causes of death."	"The expression level of C/EBP homologous protein can serve as a potential biomarker of death from mechanical asphyxia."
Ishikawa	2010	10.1016/ j.legalmed.2010.01.004	Leg Med (Tokyo)	Japan	Hypothermia and hyperthermia	Immunotechniques	290	0	96	"[To investigate] cellular immunopositivity for adrenaline, noradrenaline and dopamine in the hypothalamus, adenohypophysis and adrenal medulla with special regard to fatal hypothermia and hyperthermia in medicolegal autopsy cases."	"Hyperthermia cases showed a lower neuronal dopamine-immunopositivity in the hypothalamus than hypothermia cases, while noradrenaline- and dopamine-immunopositivities in the adrenal medulla were higher for hyperthermia than for hypothermia. Rates of noradrenaline-immunopositivity in the adrenal medulla were very low for hypothermia."
Jack	2014	10.1007/ s00381-013-2348-5	Childs Nerv Syst	US	Other	Immunotechniques	33	0	1	"In addition to determining the inflammatory cellular composition and presence of iron in foetal and infant leptomeninges associated with natural disease processes and in the absence of physical trauma beyond that accompanying vaginal birth, this study aims to formulate a basis of comparison of leptomeningeal cellular constituents in forensic settings, based on rigorous histological analyses of hospital-derived autopsies."	"CD45, CD68 and CD163 positive inflammatory cells were identified in the leptomeninges of sections of the cerebellum, brain stem and cortex of all cases of non-traumatic infant deaths. Iron was found in the leptomeninges in several cases, even those without recent haemorrhage."
Jack	2019	10.1007/ s00381-019-04268-z	Childs Nerv Syst	US	Sudden unexpected death in infancy and childhood	Immunotechniques	58	0	0	"To determine the presence and pattern of inflammatory cell distribution and iron deposition in deaths certified as sudden infant death syndrome/sudden unexpected death in infancy."	"The ranges of the number of [immunoreactive] cells per millimeter, and the standard deviations of the means were wide and varied. Overall, there was no significant difference in the number of CD45 or CD68 immunoreactive cells/millimeter between the [cerebral cortex, brain stem and cerebellum]. Comparing this cohort to a subpopulation of hospitalized infants in our prior study, there were no significant differences between the density of inflammatory cells in the sections from the cerebral cortex and brain stem. Iron was identified in only a single section in this cohort but was present in most of the cases in the hospital-based cohort."
Johnson	2012	10.1111/ j.1556-4029.2012.02212.x	J Forensic Sci	US	Substance abuse	Genetic techniques	36	20	66	"[To examine] heat shock protein 70-related gene expression in postmortem specimens from a series of cocaine-related deaths and well-matched drug-free control subjects."	"Heat shock protein 70 expression was increased significantly in cocaine abusers compared to control subjects, irrespective of the presence or absence of excited delirium. Furthermore, elevated heat shock protein 70 expression was predictive of a period of survival between cocaine use and death that included medical and/or police intervention."
Karayel	2010	10.1097/ PAF.0b013e3181c160d9	Am J Forensic Med Pathol	Turkey	Substance abuse	Macroscopic observation	17	25	69	"To investigate various central nervous system lesions of methanol intoxication in cases autopsied in the mortuary department of the Council of Forensic Medicine in Istanbul, Turkey."	"In 8 cases (47%), cerebral edema and in 9 cases (53%) at occipital, temporal and parietal cortex, basal ganglia and pons, petechial bleeding was observed. In addition to these findings, hemorrhagic necrosis were observed in thalamus, putamen, and globus pallidus in 5 cases (29.4%) and, in cerebral cortex in another 3 cases (17.6%)."
Kibayashi	2012	10.1097/ PAF.0b013e3181fe33e8	Am J Forensic Med Pathol	Japan	Traumatic intracranial injury	Several primary methods	66	0	93	"[To analyze] the forensic autopsy results of cases of fatal closed head injury to determine causal factors and the time course of development of posttraumatic pituitary lesions."	"Pituitary lesions were identified in 27 [of 66] patients. In patients with pituitary lesions, posterior lobe hemorrhage was observed in 21 patients, followed by anterior lobe hemorrhage in 10 patients and anterior lobe infarct in 7 patients. Immunohistochemistry of neurophysin showed increased immunoreactivity in the hypothalamus of patients with pituitary lesions and brain edema, providing morphologic evidence of pituitary dysfunction."
Kinney	2015	10.1007/ s00401-014-1357-0	Acta Neuropathol	US	Sudden unexpected death in infancy and childhood	Several primary methods	153	0	1	"[To test the hypothesis that] a well-established marker of hippocampal pathology in temporal lobe epilepsy—focal granule cell bilamination in the dentate, a variant of granule cell dispersion—is associated with sudden unexplained death in infants."	"Focal granule cell bilamination was present in 41.2% of the unexplained group compared to 7.7% of the explained (control) group. It was associated with a cluster of other dentate developmental abnormalities that reflect defective neuronal proliferation, migration, and/or survival."
Kobek	2016	10.5114/ fn.2016.58915	Folia Neuropathol	Poland	Traumatic intracranial injury	Immunotechniques	100	N/a	N/a	"To analyze morphometrically changes in neurofilaments following the brain contusion and relate them to the length of the time of survival."	"There are significant differences in numbers and area fractions of neurofilaments within 7 days after head trauma."
Krohn	2015	10.1089/ neu.2014.3524	J Neurotrauma	Germany	Traumatic intracranial injury	Immunotechniques	57	18	85	"To determine whether the percentage of S100 protein and neuronal specific enolase positivity in neuroglial cells, as well as in neurons of different brain regions, were associated with the cause of death and the survival time after traumatic brain injury."	"The percentages of S100-positive oligodendrocytes in the pericontusional zone in cases with subacute death after brain injury were significantly lower than in controls and in the acute death after brain injury group. In the hippocampus, S100-positive oligodendrocytes were significantly lower in cases with acute death after brain injury and subacute death after brain injury, compared with controls. It is of particular interest that there were also S100-positive neurons in the pericontusional zone and hippocampus in traumatic brain injury cases after more than 2 h survival but not in acute death after brain injury cases or controls. In conclusion, the present findings emphasize that S100 and neuronal specific enolase immunopositivity might be useful for detecting the cause and process of death due to traumatic brain injury."
Krywanczyk	2018	10.1111/ 1556-4029.13634	J Forensic Sci	US	Traumatic intracranial injury	Immunotechniques	17	0	4	"[To describe] the quantity and distribution of macrophages and hemosiderin-laden macrophages [in children under the age of 5 without a history of head trauma, without macroscopically apparent subdural hemorrhage, and without macroscopic or microscopic neomembrane]."	"CD68-positive cells were present in all cases, even in the dural border layer. Iron-containing cells were identified in 59% of cases, and in the dural border layer in 29%. Therefore, CD68-positive and iron-containing cells can be present in pediatric dura without neomembrane or macroscopic subdural hemorrhage, and this requires consideration when estimating the age of a subdural hematoma."
Krzyżanowska	2016	10.1007/ s00406-015-0655-4	Eur Arch Psychiatry Clin Neurosci	Poland	Suicide	Genetic techniques	57	N/a	N/a	"[To test the hypothesis of] a decreased ribosomal DNA transcriptional activity in dorsal raphe nucleus neurons in suicide completers regardless of their underlying psychiatric diagnosis."	"Significant decreases in silver-stained nucleolar organising region parameters suggestive of attenuated ribosomal DNA activity were found in the cumulative analysis of all dorsal raphe nucleus subnuclei in suicide victims versus controls."
Kurtulus Dereli	2018	10.1177/ 0025802418797178	Med Sci Law	Turkey	Suicide	Immunotechniques	42	18	40	"[To evaluate] the relationship between pinealocyte acetylserotonin O-methyltransferase-immunoreactivity and suicide."	"The acetylserotonin O-methyltransferase-immunopositive pinealocyte count was observed to be lower in suicide cases compared to the non-suicide cases. These results support decreased pineal gland activity in suicide."
Lech	2011	10.1007/ s12011-010-8747-5	Biol Trance Elem Res	Poland	Other	Other	48	14	80	"[To study] the concentration of zinc in human tissues of normal subjects in Poland."	"The following values were found by the flame atomic absorption method (mean ± SD, median, range, in microgram per gram): brain 10.3 ± 1.36, 10.2, 7.99–13.8 (n=48)."
Leitner	2022	10.1111/ nan.12746	Neuropathol Appl Neurobiol	US	Sudden unexpected death in infancy and childhood	Histology and conventional staining	45	0	10	"To determine the frequency of hippocampal findings in sudden unexplained death in childhood cases compared with age-matched sudden explained death in childhood control cases."	"Neither sudden unexplained death in childhood (51.2%) nor control (55.9%) slides were considered contributory to determining cause of death. The lack of an association of hippocampal findings in sudden unexplained death in childhood and controls, as well as inconsistency of observations by multiple blinded reviewers, indicates an inability to reliably identify hippocampal maldevelopment associated with sudden death."
Lesnikova	2018	10.1097/ PAF.0000000000000408	Am J Forensic Med Pathol	Demark	Postmortem interval	Immunotechniques	40	N/a	N/a	"To assess the importance of postmortem interval time on the accuracy of immunohistochemistry-based autopsy diagnoses."	"An overall correlation between the postmortem interval and the immunohistochemistry score for all tissue samples was found. Samples from decedents with a postmortem interval of 1 to 3 days showed positive staining with all antibodies, whereas samples from decedents with a longer postmortem interval showed decreased staining rates."
Liang	2020	10.1007/ s00414-019-02169-z	Int J Legal Med	China	Other	Several primary methods	13	0	13	"To determine the pathological changes that resulted from exposure to phosphine and, secondly, to determine whether oxidative stress was involved in phosphine-induced neurotoxicity using histopathological and immunohistochemistry methods."	"We found severe damage induced by phosphine in many systems, especially the neurological system, including neuronal, axonal, and vascular injuries as well as oxidative damage, which indicated that oxidative stress was a crucial mechanism for neuronal death in phosphine toxicity."
Lier	2020	10.1007/ s00414-020-02308-x	Int J Legal Med	Germany	Traumatic intracranial injury	Immunotechniques	13	18	78	"To determine acute changes in microglial morphology and antigen expression after traumatic brain injury."	"We detected highly localized changes in microglial morphology already early after traumatic damage, e.g., activated microglia and phagocyted erythrocytes in the contusion areas in cases with minute survival. Furthermore, an altered antigen expression was observed with increasing trauma wound age."
Lindenbergh	2013	10.1007/ s00414-013-0895-7	Int J Legal Med	Netherlands	Brain tissue identification	Genetic techniques	19	N/a	N/a	"[To] examine a total of 41 candidate messenger RNA markers for their ability to differentiate between brain, lung, liver, skeletal muscle, heart, kidney and skin."	"14 markers are regarded tissue-specific and included in an endpoint reverse transcription quantitative polymerase chain reaction multiplex together with one general muscle, one blood and one house-keeping marker. This 17-plex is successfully used to analyse a blind test set of 20 specimens including mixtures, and samples derived from stabbing of organ tissues."
Lundesgaard Eidahl	2019	10.1016/ j.jflm.2019.03.003	J Forensic Leg Med	Norway	Brain edema	Conventional measurements (e.g. weight)	54	4	89	"[To test the hypothesis that] brains with edema have increased water content compared to nonedema cases."	"[There was] a significant relationship between brain weight and inner skull circumference, with the ratio between these two parameters being significantly higher in cases with severe postmortem brain edema than in cases with very little or no brain edema."
Matoba	2017	10.1016/ j.legalmed.2017.01.005	Leg Med (Tokyo)	Japan	Laboratory methods and quality	Macroscopic observation	20	20	88	"To evaluate the usefulness of the applied freezing technique in putrefied brain for macroscopic investigation."	"In the freezing group, the entire putrefied brain was extracted as a solid organ, the gray-white matter differences were well visible. The freezing procedures to evaluate the putrefied brain have been successfully applied, and it could be statistically more useful in putrefied brain investigation than the ordinary procedure."
Mehrpour	2010	10.1016/ j.jflm.2010.08.012	J Forensic Leg Med	Iran	Anatomy	Conventional measurements (e.g. weight)	1143	16	88	"To formulate a standard reference range of brain weight, taking into account the variables of age, sex, weight, body mass index and height."	"Brain weight in males and females was 1322.45 ± 117.05 and 1219.03 ± 127.85 g respectively. A negative significant correlation between age and brain weight was seen in both sexes. Moreover, there was a positive significant correlation between body height and brain weight in males and females."
Mohd Saman	2021	10.1097/ PAF.0000000000000639	Am J Forensic Med Pathol	Malaysia	Traumatic intracranial injury	Macroscopic observation	126	N/a	N/a	"[To describe] motorcycle casualties in Malaysia."	"The most common injury [was] intracranial hemorrhage (74%)."
Molina	2020	10.1111/ 1556-4029.14174	J Forensic Sci	US	Substance abuse	Conventional measurements (e.g. weight)	246	16	69	"To see whether the old adage of using the triad of cerebral and pulmonary edema and bladder fullness to suggest an opioid death could be used to differentiate deaths due to opioid toxicity from deaths due to cardiac disease."	"Opioid-related deaths were more likely to have a heavy brain."

First author	Year	DOI	Journal	Location[1]	Primary theme[2]	Primary method[3]	N[4]	Age[5] (years)		Aims (quoted[6])	Main findings/conclusions (quoted[6])
								Min	Max		
Mori	2018	10.1016/ j.jstrokecerebrovasdis. 2017.09.031	J Stroke Cerebrovasc Dis	Japan	Other	Macroscopic observation	607	20	93	"[To perform] a statistical analysis of medicolegal autopsy cases involving ruptured intracranial aneurysms and dissections in terms of their pathological and epidemiological features."	"Aneurysms were found to occur much more frequently in the anterior communicating artery (31.9%) and vertebral arteries (7.5%), while dissections were found much more commonly in vertebral arteries (93.7%). The size of aneurysms was much smaller in general than that previously regarded as a risk factor of rupturing."
Mubbunu	2018	10.1155/ 2018/4687538	Anat Res Int	Zambia	Anatomy	Conventional measurements (e.g. weight)	114	16	85	"To correlate the weight of internal organs with body weight and height."	"The brain [was] positively correlated to body weight [and] height in the male population. In the female population, the brain [was] positively correlated to the weight of the body."
Naue	2018	10.1016/ j.fsigen.2018.07.007	Forensic Sci Int Genet	Germany	Other	Genetic techniques	29	0	87	"[To investigate] the age dependency of 13 loci in DNA obtained from brain."	"Seven of these loci did show age-dependency. This pilot study shows the potential of existing blood DNA methylation markers for age-determination to analyze other tissues than blood."
Nishida	2015	10.1111/ nan.12229	Neuropathol Appl Neurobiol	Japan	Neurodegeneration	Several primary methods	887	0	101	"To detect preclinical or early clinical corticobasal degeneration cases by examining a series of forensic autopsy cases."	"Three autopsy cases (0.34%) were identified that fulfilled corticobasal degeneration pathological criteria. Significant microscopic differences between the subclinical and clinical cases included occurrence of neuronal loss with spongiosis and gliosis, as well as a difference in degree of tau pathology in the superficial layer of the neocortical areas and white matter. Immunohistochemistry is essential for detecting preclinical corticobasal degeneration cases."
Nyasa	2021	10.5603/ FM.a2020.0142	Folia Morphol (Warsz)	Malawi	Anatomy	Macroscopic observation	24	3	65	"To understand and describe the anatomy of the circulus arteriosus cerebri among indigenous Malawians."	"The complete-circle configuration was found in 69.57% of the circuli arteriosi cerebri. Of these, 37.5% were typical, representing an overall typicality prevalence of 26.09%. Vessel asymmetry was observed in 30.43% of cases."
Oerter	2019	10.1007/ s00414-018-1893-6	Int J Legal Med	Germany	Traumatic intracranial injury	Immunotechniques	19	15	95	"[To focus on] the pathomechanism of brain glucose supply via sodium/glucose cotransporters 1 and 2 following traumatization."	"The immunoreactivity in contusional cerebral cortex region began to increase 3 to 7 h following traumatization. Both sodium/glucose cotransporter 1 and 2 protein expression increased significantly 37 h post-injury compared to the control group. Sodium/glucose cotransporter 1 and 2 protein expression may be useful in forensic practice as an effective target to analyze the existence of a traumatic brain injury and to determine the time of the traumatization."
Olczak	2018	10.1007/ s12024-017-9942-x	Forensic Sci Med Pathol	Poland	Asphyxia and hypoxia	Immunotechniques	24	N/a	N/a	"To examine if bystin might be a useful marker for hypoxic-ischemic changes in forensic cases."	"Groups suspected of acute hypoxic-ischemic changes presented strong bystin expression in the cytoplasm of neocortical neurons, that seemed to be short-lasting. In the hypoxic-ischemic-reperfusion group we did not find bystin expression. Bystin expression in the cytoplasm of cortical neurons was minimal in the control group (cardiac arrest)."
Olczak	2019	10.5114/ fn.2019.83831	Folia Neuropathol	Poland	Traumatic intracranial injury	Immunotechniques	38	N/a	N/a	"To preliminarily evaluate and qualitatively describe the expression of dynein, dynactin, and kinesin by performing immunohistochemical staining within the brain tissue in postmortem examination."	"We documented that dynein, dynactin, and kinesin staining should be considered as a supplemental diagnostic tool for traumatic brain injury in postmortem neuropathological examination and forensic autopsy."
Pelletier	2017	10.23907/ 2017.011	Acad Forensic Pathol	US	Substance abuse	Macroscopic observation	150	20	40	"To highlight frequent findings of opioid overdoses."	"Cerebral edema occurred in 54% of decedents who died of opiates/opioids excluding fentanyl and 8% in those who died solely of fentanyl. The control group found 2% had cerebral edema."
Pelletti	2019	10.1016/ j.forsciint.2019.01.025	Forensic Sci Int	Italy	Postmortem interval	Other	4	N/a	N/a	"1. To develop and validate a gas chromatography–mass spectrometry method for the determination of Putrescine and Cadaverine on human brain samples (phase 1 - Validation study);<br>2. To study the relation of Putrescine and Cadaverine concentration and the postmortem interval on 12 human brain aliquots along a period of decomposition (120h) under experimental conditions (phase 2 - Decomposition study)."	"Validation study: Both Putrescine and Cadaverine validation parameters were within the acceptable values, with better selectivity, linearity, accuracy and precision values for Putrescine.<br>Decomposition study: A significant relationship between Putrescine and Cadaverine levels and postmortem interval has been demonstrated with a correlation coefficient of 0.98 for Putrescine and 0.93 for Cadaverine."
Preusse-Prange	2014	10.1016/ j.forsciint.2013.12.004	Forensic Sci Int	Germany	Laboratory methods and quality	Immunotechniques	6	46	67	"To find out if the formalin fixation time leads to different results in detection and quantification of proteins from the heat shock protein 70 family."	"Western blot analysis of formalin fixed tissues does not allow a reliable detection of proteins at all, while a reproducible detection by immunohistochemistry was still possible after one month of incubation."
Priemer	2021	10.1093/ jnen/nlab059	J Neuropathol Exp Neurol	US	Neurodegeneration	Several primary methods	78	50	106	"To report the utility of the protocol [for the neuropathologic assessment of Alzheimer's disease and other dementias] when actively applied to forensic neuropathology practice, and more largely to survey the diversity and distribution of neurodegenerative diseases in the forensic setting."	"[At least one] neurodegenerative disease was identified in 94.9% [of the] brains; the most common were Alzheimer's disease (52.5%), primary age-related tauopathy (33.3%), and Lewy body disease (32.1%). 89.7% [of the] cases had chronic cerebrovascular disease."
Rao	2016	10.7860/ JCDR/2016/ 19783.8141	J Clin Diagn Res	India	Traumatic intracranial injury	Histology and conventional staining	100	N/a	N/a	"[To correlate] histopathological changes following subdural haemorrhages with known post-traumatic intervals in medico legal perspective."	"All the histomorphological features, when correlated with post-traumatic interval groups, were found to be statistically significant, except for polymorphonuclear leukocytes. Routine histopathology was reliable in the dating of early subdural haemorrhages."
Rebollo-Soria	2016	10.1016/ j.jflm.2016.07.009	J Forensic Leg Med	Spain	Traumatic intracranial injury	Macroscopic observation	35	16	92	"To describe the injury pattern of pedestrians who have been struck by motorcycles that resulted on death, in urban areas."	"Traumatic brain injury is the main cause of death in pedestrian hit by motorized two-wheeled vehicles (62.85%). The most frequent injury was the subarachnoid hemorrhage, in 71.4% of cases, followed by cerebral contusions and skull base fractures (65.7%)."
Romero Tirado	2018	10.1097/ PAF.0000000000000412	Am J Forensic Med Pathol	Spain	Traumatic intracranial injury	Immunotechniques	25	33	94	"To study the immunocytochemical structural (neurofilament antibody) and functional (β-amyloid precursor protein) response to axonal injury as an expression of cortical cerebral damage, comparing cases in which death had nothing to do with central nervous system injury with other cases in which death was secondary to head trauma."	"The neurofilament antibody shows beaded axons since the first moment; over time, they increase their density and diameter as survival time also increases. These changes begin in the gray matter, 2 hours after trauma can be seen around vessels and in hemorrhagic areas. At 24 hours, beaded axons appear in the white mater, which finally loses its structure and cellular density. The β-amyloid precursor protein marker begins to be weakly seen 2 hours after injury. At 24 hours, a diffuse pattern can appear."
Rungruangsak	2021	10.1016/ j.jflm.2021.102226	J Forensic Leg Med	Thailand	Traumatic intracranial injury	Macroscopic observation	862	N/a	N/a	"To describe the brain pathology of severe head injuries with lethal outcomes in terms of the incidence of macroscopic lesions (diffuse vascular injury, coup-contrecoup contusions, and cerebral edema) as well as microscopic lesions (diffuse axonal injury)."	"The hallmarks of severe traumatic brain injury—such as a period for detecting diffuse axonal injury via conventional staining—were evident. However, it was noticed that the histopathological detection rates of diffuse axonal injury surged after 72 h, which might be because these injuries are mediated by secondary axotomy. The increment time of cerebral edema reached a peak in 12 h, after which the condition sustained for at least 72 h."
Sadat-Shirazi	2018	10.1016/ j.neulet.2018.09.043	Neurosci Lett	Iran	Substance abuse	Several primary methods	69	20	51	"[To evaluate] dopamine receptor expression level in the brain of opioid abusers in comparison with the control group."	"The messenger RNA and protein level of dopamine receptor type 1 increased in the ventral tegmental area, nucleus accumbens and amygdala of opioid abusers. Dopamine receptor type 2 protein level increased in the ventral tegmental area, nucleus accumbens and amygdala of opioid abusers when compared with the control. Dopamine receptor type 3 level decreased in all the brain regions except in the amygdala of opioid abusers in comparison with the control group. Dopamine receptor type 4 messenger RNA level increased only in the amygdala of opioid abusers. In messenger RNA and protein level of dopamine receptor type 5, it followed the same pattern like dopamine receptor type 1."
Sadat-Shirazi	2020	10.1016/ j.jocn.2019.12.064	J Clin Neurosci	Iran	Substance abuse	Immunotechniques	114	N/a	N/a	"To measure malondialdehyde level, super oxide dismutase activity and cyclooxygenase 2 protein level in the prefrontal cortex [among pure-opioid and multi-drug abusers compared to controls]."	"The level of malondialdehyde was increased in the medial prefrontal cortex, lateral prefrontal cortex and orbitofrontal cortex of pure-opioid and multi-drug abusers compared with the control group. The super oxide dismutase activity was reduced in the medial prefrontal cortex, lateral prefrontal cortex and orbitofrontal cortex of abusers in comparison to the control group. The protein level of cyclooxygenase 2 was decreased in the medial prefrontal cortex and lateral prefrontal cortex of multi-drug abusers compared with the control group."
Sadat-Shirazi	2020	10.1016/ j.npep.2020.102074	Neuropeptides	Iran	Substance abuse	Immunotechniques	114	N/a	N/a	"[To examine] the level of protein kinase Cα in the amygdala, nucleus accumbens, medial prefrontal cortex, orbitofrontal cortex, and lateral prefrontal cortex [among pure-opioid and multi-drug abusers compared to controls]."	"The level of protein kinase Cα increased in the prefrontal cortex and amygdala of the abusers compared with the control group, although we did not detect changes in the level of protein kinase Cα in the nucleus accumbens."
Sakai	2014	10.1097/ PAF.0000000000000067	Am J Forensic Med Pathol	Japan	Traumatic intracranial injury	Immunotechniques	36	1	89	"To analyze how the ubiquitin proteasome system and autophagy are induced in brain tissues at different intervals after traumatic intracranial injury."	"The number of neurons and glial cells with cytoplasmic inclusions that stained positive for ubiquitin, lysine 48-linked polyubiquitin chains, and protein p62 began to increase within 1 hour after intracranial injury, particularly at contusion sites. From 3.5 hours onward, an increase in cytoplasmic inclusions that stained positive for lysine 63-linked polyubiquitin chains and microtubule-associated protein 1 light chain 3 began to be detected."
Samsuwan	2018	10.1016/ j.forsciint.2018.02.032	Forensic Sci Int	Thailand	Brain tissue identification	Genetic techniques	15	N/a	N/a	"[To develop] a technique for identifying brain tissue in aged samples [using the Combined Bisulfite Restriction Analysis technique]."	"Combined Bisulfite Restriction Analysis—for EML2 (COBRA-EML2) technique was established and validated. COBRA-EML2 displayed 100% sensitivity and specificity for distinguishing brain tissue from other tissues, showed high reliability, was capable of detecting minimal DNA concentration, could be used for identifying brain tissue in aged samples."
Sauer	2017	10.1016/ j.fsigen.2017.02.002	Forensic Sci Int Genet	Germany	Brain tissue identification	Genetic techniques	N/a	N/a	N/a	"To employ micro-RNA markers for the identification of brain, kidney, liver, lung, skin, heart muscle and skeletal muscle in a forensic context."	"We show that not only can micro-RNA expression profiling be used to reliably differentiate between organ tissues but also that this method, which is compatible with and complementary to forensic DNA analysis, is applicable to realistic forensic samples e.g. mixtures, aged and degraded material as well as traces generated by mock stabbings and experimental shootings at ballistic models."
Schober	2015	10.1007/ s00414-014-1129-3	Int J Legal Med	Germany	Traumatic intracranial injury	Genetic techniques	15	19	80	"To determine the influence of hypoxia on traumatic brain injuries [by conducting a gene expression analysis]."	"From a total of 667 microRNAs, altogether, 248 microRNAs appeared expressed with 13 of them showing significant differences in the mean gene expression. The combination of two messenger RNAs (HSPA12B/FOSB or IL6/HSD11B1) or two microRNAs (either miR-138/miR-744 or miR-195/miR-324-5p) completely discriminated [between individuals who had died from severe frontal cortex injuries or due to natural causes], a finding unaltered by age at biosampling, survival time, and the postmortem interval."
Shaha	2010	10.1258/ msl.2010.010008	Med Sci Law	India	Other	Macroscopic observation	118	N/a	N/a	"To investigate the epidemiology of electrical fatalities occurring in Coimbatore."	"The most common cause of death was cardiac arrest, followed by septicaemia and renal failure. Congestion of the brain and oedematous lungs were frequent non-specific postmortem findings."

First author	Year	DOI	Journal	Location[1]	Primary theme[2]	Primary method[3]	N[4]	Age[5] (years)		Aims (quoted[6])	Main findings/conclusions (quoted[6])
								Min	Max		
Sheikhazadi	2010	10.1016/ j.jflm.2009.07.012	J Forensic Leg Med	Iran	Anatomy	Conventional measurements (e.g. weight)	1222	15	88	"[To] examine the normal adult internal organ weight and its relationship with body height, body weight, body mass index and age."	"Organ weights decreased with age except for the heart and the prostate, and increased in relation to body height and/or body mass index. Except for the brain, the organ weight showed a better statistical correlation with the body mass index than the body height."
Siddiqi	2013	N/a	J Coll Physicians Surg Pak	Pakistan	Anatomy	Macroscopic observation	51	20	70	"To find the variations in the anatomy of the arterial circle of Willis in the adult Pakistani population."	"[Of the 51 cerebral arterial circles,] 29.4% had typical configuration; 49% had symmetrical arrangement and 76.4% had different types of variations in their component vessels. Variations were most common in the posterior communicating artery."
Sultana	2018	N/a	Mymensingh Med J	Bangladesh	Anatomy	Macroscopic observation	60	20	59	"To study the incidence of exact location & diameter of basilar artery to see the variation with age in Bangladeshi people."	"The mean diameter of basilar artery gradually decreased with increase of age at all levels & the diameter of artery was found narrowest at its mid level."
Takayama	2016	10.1016/ j.legalmed.2016.08.007	Leg Med (Tokyo)	Japan	Neurodegeneration	Immunotechniques	48	N/a	N/a	"To develop of a convenient, prompt retrieval method for dementia that uses immunohistochemical staining instead of silver staining."	"Differences in beta-amyloid-positive senile plaques and tau-immunopositive neurofibrillary tangles were effective for discriminating between [the dementia group and the non-dementia group]. These criteria may reveal the presence and progression of dementia."
Takayama	2016	10.2152/ jmi.63.114	J Med Invest	Japan	Neurodegeneration	Other	14	N/a	N/a	"To quantify pathological findings for the diagnosis of dementia using image analysis software."	"An easy, simple, and effective quantification method of the pathological findings was achieved. However, no significant differences were observed between [the dementia group and the non-dementia group], and diagnosis of dementia by the quantification of pathological findings was not successful."
Tolescu	2020	10.47162/ RJME.61.1.10	Rom J Morphol Embryol	Romania	Traumatic intracranial injury	Macroscopic observation	1005	N/a	N/a	"[To evaluate] the deaths caused by traumatic brain injuries investigated in a Department of Forensic Medicine in Romania – the Institute of Forensic Medicine of Craiova –, over a period of seven years."	"78% of the adults and 44.12% of children presented subdural hematomas associated with other meningo-cerebral lesions. Also, 83.63% of the adults and 97% of children presented brain contusions. In both groups, brain laceration was observed in approximately 50% of the cases."
Tong	2017	10.1016/ j.forsciint.2017.06.015	Forensic Sci Int	China	Other	Immunotechniques	4	27	86	"To investigate the pathologic changes resulting from insulin overdose in a series of established homicidal insulin overdose cases using hematoxylin and eosin, immunohistochemistry, and immunofluorescence assays."	"Severe reactive astrocyte proliferation was obvious in the white matter of the cerebrum, corpus callosum, cerebellum and brain stem, especially in subcortical regions."
Trautz	2018	10.1007/ s00414-017-1767-3	Int J Legal Med	Germany	Laboratory methods and quality	Immunotechniques	N/a	N/a	N/a	"To assess the optimal approach for different technical aspects of immunohistochemistry, in order to improve and standardize this procedure."	"Automatic staining showed more consistent staining results, compared to manual staining procedures. Digitalization and digital post-processing facilitated direct analysis and analysis for reproducibility considerably. No differences were found for different commercially available microscopic glass slides regarding suitability of immunohistochemistry brain researches, but a certain rate of tissue loss should be expected during the staining process."
Trautz	2019	10.1038/ s41598-019-48145-w	Sci Rep	Germany	Traumatic intracranial injury	Immunotechniques	75	18	91	"[To compare] the immunohistochemical and immunofluorescence profile for intracellular interleukin-6 and glial fibrillary acidic protein in brain tissue between lethal traumatic brain injury (with well documented survival time) and cardiovascular causes of death, to determine whether the level of positive reactivity of both proteins in neurons or glial cells of various brain regions is associated with the cause of death and the survival time after fatal traumatic brain injury."	"Interleukin-6 positive neurons, glial cells and glial fibrillary acidic protein positive astrocytes all concordantly increase with longer trauma survival time, with statistically significant changes being evident from three days post-traumatic brain injury in the pericontusional zone, irrespective of its definite cortical localization. Interleukin-6 staining in neurons increases significantly in the cerebellum after trauma, whereas increasing glial fibrillary acidic protein positivity is also detected in the cortex contralateral to the focal lesion. These systematic chronological changes in biomarkers of pericontusional neurons and glial cells allow for an estimation of trauma survival time."
van den Berge	2016	10.1007/ s00414-016-1438-9	Int J Legal Med	Netherlands	Postmortem interval	Genetic techniques	13	N/a	N/a	"[To] apply various DNA analysis techniques and messenger RNA profiling to search for trends relating nucleic acid degradation and postmortem interval in exhumed human organ tissues (i.e. brain, lung, liver, skeletal muscle, heart, kidney and skin samples) with burial times ranging from 4 to over 42 years."	"Brain and heart are the organs in which both DNA and RNA remain remarkably stable, notwithstanding long postmortem intervals. No straight-forward relations were observed between nucleic acid profiling results and the postmortem interval."
Vasovic	2012	10.1007/ s00701-012-1400-7	Acta Neurochir	Serbia	Anatomy	Macroscopic observation	216	0	95	"[To study] vertebrobasilar dolichoectasia in human cadavers."	"There were 14 cases [of 216] of vertebral and/or basilar (dolicho)ectasia. The basilar length ranged from 32.91–59.37 mm, and the basilar outer diameter ranged from 3.51–8.92 mm in relation to the corresponding point of its measurement. The outer diameter of the vertebral arteries ranged from 0.67–5.91 mm."
Wang	2012	10.1007/ s00414-012-0774-7	Int J Legal Med	Japan	Laboratory methods and quality	Genetic techniques	15	16	79	"[To calculate] amplification efficiencies in quantitative real-time reverse transcriptase polymerase chain reaction for 32 potential reference genes in postmortem human brain tissues using a completely objective and noise-resistant algorithm to select candidate reference genes."	"24 genes met standard efficiency criteria. Combining three algorithms suggested the genes IPO8, POLR2A, and PES1 as stable endogenous references in quantitative real-time reverse transcriptase polymerase chain reaction analysis of human brain samples, with YWHAZ, PPIA, HPRT1, and TBP being the least stable ones."
Wang	2012	10.1016/ j.forsciint.2012.01.015	Forensic Sci Int	Japan	Hypothermia and hyperthermia	Immunotechniques	72	21	92	"[To immunohistochemically investigate] basic fibroblast growth factor, glial fibrillary acidic protein, S100 calcium-binding protein B and single-stranded DNA in the brains of fatalities due to extreme environmental temperatures involving hypothermia and hyperthermia for evaluating central nervous system dysfunction."	"Characteristic findings in hypothermia cases were higher glial basic fibroblast growth factor immunopositivity in the cerebral cortex and white matter, and higher S100 calcium-binding protein B immunopositivity in the cerebral cortex. Hyperthermia showed lower glial factor, glial fibrillary acidic protein and S100 calcium-binding protein B immunopositivities in the white matter, and higher neuronal single-stranded DNA immunopositivity in the cerebral cortex and hippocampus, accompanied by high glial basic fibroblast growth factor and S100 calcium-binding protein B immunopositivities in the cerebral cortex."
Wang	2012	10.1016/ j.forsciint.2012.04.025	Forensic Sci Int	Japan	Traumatic intracranial injury	Immunotechniques	198	1	97	"[To immunohistochemically investigate] the expressions of basic fibroblast growth factor and glial fibrillary acidic protein in the glial cells as well as single-stranded DNA positivity in the neurons at sites distant from primary injury to detect the survival time-dependent changes in forensic autopsy cases of fatal traumatic brain injury in relation to parahippocampal herniation or secondary brainstem hemorrhage of Duret as a macroscopic sign of brain swelling and compression."	"The combined use of basic fibroblast growth factor, glial fibrillary acidic protein and single-stranded DNA immunohistochemistry can be used to evaluate the severity of damage and response of brain after traumatic brain injury."
Wang	2013	10.1007/ s00414-013-0868-x	Int J Legal Med	Japan	Other	Several primary methods	101	17	97	"[To analyze] the gene expressions of aquaporin 1, aquaporin 4, claudin 5, matrix metalloproteinase 2, and matrix metalloproteinase 9 in the brains of forensic autopsy cases, combined with immunohistochemical detections, to investigate the molecular pathology of brain edema in fire fatalities with special regard to the importance of reference gene selection."	"Prolonged deaths due to severe burns showed an increase in brain water content, but relative messenger RNA quantification, using different normalization methods, showed inconsistent results. In immunostaining, only aquaporin 1 and matrix metalloproteinase 9 showed differences among the causes of death: they were evident in most prolonged deaths due to severe burns."
Wang	2014	10.1007/ s00414-014-0972-6	Int J Legal Med	Japan	Substance abuse	Several primary methods	114	18	90	"[To analyze] the gene expressions of matrix metalloproteinase 2, matrix metalloproteinase 9, claudin 5, aquaporin 1 and aquaporin 4, using reverse transcription quantitative polymerase chain reaction, combined with immunohistochemical detections, to investigate the molecular pathology in the brains of forensic autopsy cases with special regard to methamphetamine intoxication."	"Relative messenger RNA quantification demonstrated higher expression of aquaporin 4 and matrix metalloproteinase 9, lower expression of claudin 5 in methamphetamine intoxication cases and lower expression of matrix metalloproteinase 2 in phenobarbital intoxication cases. Immunostaining results showed substantial interindividual variations in each group, showing no evident differences in distribution or intensity among all the causes of death."
Wong	2015	10.1016/ j.jflm.2014.09.012	J Forensic Leg Med	Australia	Traumatic intracranial injury	Macroscopic observation	12	22	48	"To assess if the sites of ruptured vessels [in cases of traumatic basal subarachnoid haemorrhage] were identified and if identified, by which methods (visual, angiography or histology)."	"Vascular injury was identified in eight of the twelve cases, all of which occurred intra-cranially, with seven involving the vertebral artery. Histology was most reliable in identifying the rupture site."
Yarid	2015	10.1111/ 1556-4029.12838	J Forensic Sci	US	Other	Macroscopic observation	27	0	87	"To examine the assertion that globus pallidus necrosis is typical of carbon monoxide poisoning, this study examined autopsycases from the King County Medical Examiner's Office between 1994 and 2013."	"Twenty-seven cases with bilateral basal ganglia lesions were identified and examined for associated or causative disease or injury. [There was] no support for the assertion that globus pallidus necrosis is characteristic of carbon monoxide poisoning."
Yoshida	2011	10.1007/ s00414-009-0374-3	Int J Legal Med	Japan	Hypothermia and hyperthermia	Immunotechniques	298	0	97	"[To investigate cellular cromogranin A immunopositivity in the hypothalamus and hypophysis] in serial medicolegal autopsy cases to examine the feasibility of using cromogranin A as a marker with special regard to fatal hypothermia and hyperthermia."	"Cellular cromogranin A immunopositivity in the hypothalamus [and] adenohypophysis varied extensively. However, cromogranin A immunopositivity in hypothalamus neurons was lower for hypothermia than other causes of death."
Yoshida	2017	10.1007/ s00401-016-1665-7	Acta Neuropathol	Japan	Neurodegeneration	Immunotechniques	29	64	94	"[To] examine serial forensic autopsy cases, applying immunohistochemistry to detect incipient progressive supranuclear palsy and reveal the clinicopathological features of such cases."	"A total of 29 cases fulfilled the pathological criteria for progressive supranuclear palsy. All had neuronal and glial inclusions in the basal ganglia and brainstem. However, 13 cases had low tau pathology and were categorized as atypical progressive supranuclear palsy. In addition to progressive supranuclear palsy pathology, multiple types of astrocytic inclusions and comorbid proteinopathies were found."
Yoshida	2019	10.3233/ JAD-190196	J Alzheimers Dis	Japan	Neurodegeneration	Immunotechniques	189	13	39	"To explore the prevalence of cases with Alzheimer's disease-related pathology in subjects <40 years of age and to explore the association of such pathology, neuropsychiatric symptoms (particularly suicide attempts), and APOE genotype."	"Tau pathology was detected in 135 of the [189] cases. The prevalence of thread pathology was higher than that of cellular inclusions. Amyloid-β deposition was found in only 7 [of 189] cases. Alzheimer's disease-related pathology was not associated with suicide."
Zedler	2014	10.1007/ s00414-013-0913-9	Int J Legal Med	Germany	Suicide	Conventional measurements (e.g. weight)	198	19	91	"To ascertain the question whether the brain weight is significantly different between suicide victims and other death cases."	"No significant differences in brain weight were found in suicide victims compared to those of the control group."
Zhang	2013	10.1016/ j.scijus.2012.11.005	Sci Justice	China	Laboratory methods and quality	Genetic techniques	40	N/a	N/a	"To certify suitable markers in nine candidates in human tissues [e.g. brain] for data normalisation with consideration of the influence of parameters (postmortem interval and cause of death)."	"[Of the nine endogenous markers tested,] U6, GAPDH and 18S rRNA were suitable in our set of samples in various corpse conditions."

First author	Year	DOI	Journal	Location[1]	Primary theme[2]	Primary method[3]	N[4]	Age[5] (years)		Aims (quoted[6])	Main findings/conclusions (quoted[6])
								Min	Max		
Zhang	2017	10.1080/ 20961790.2017.1280890	Forensic Sci Res	China	Substance abuse	Immunotechniques	56	20	82	"Astrocytic morphological changes in the cerebral cortex of methamphetamine abusers were quantitatively analysed to investigate the neuropathological effects of methamphetamine abuse in humans."	"Clasmatodendrotic astrocytes (including those with swollen cell bodies and disintegrating distal processes) were frequently observed in the cerebral cortex of methamphetamine abusers. Quantitative analysis showed a concomitant increase in the astrocyte area and astrocyte-to-vessel area ratio in the grey matter in acute methamphetamine fatality and other methamphetamine-involved cases, although the astrocyte area wasalso increased in cases of asphyxiation."
Zhuo	2012	10.1016/ j.forsciint.2012.08.024	Forensic Sci Int	US	Sudden unexpected death in epilepsy	Macroscopic observation	74	14	63	"To re-evaluate the characteristics of postmortem examination findings of sudden unexpected death in epilepsy death in cases investigated by a statewide medical examiner's office."	"[Of 104 sudden unexpected deaths directly or indirectly caused by an epilepsy/seizure disorder], 74 cases met a general accepted definition of sudden unexpected death in epilepsy death. 58.1% showed neuropathological lesions."
Zorila	2017	N/a	Rom J Morphol Embryol	Romania	Traumatic intracranial injury	Several primary methods	29	N/a	N/a	"[To perform] an analysis of the death cases caused by traumatic brain injuries in 29 children aged less than 18 years, undergoing autopsy within the Forensic Institute of Craiova, Romania, between 2011–2016, associated with studies of microscopy and immunohistochemistry for a complete and accurate histopathological diagnosis."	"In 93% of the cases, there were complex meningo-cerebral injuries: leptomeningeal hemorrhage associated with brain contusion injuries and with intraventricular blood flood, as well as destructive lesions of brain dilaceration. The histopathological and immunohistochemical examinations showed that the severity of traumatic brain injuries increased according to the survival time, by adding secondary lesions caused by brain ischemia and local inflammatory reaction."
Zwirner	2019	10.1038/ s41598-019-52836-9	Sci Rep	Germany	Anatomy	Other	75	2	94	"To provide basic biomechanical data of human temporal dura mater obtained from [75] cadavers in a fresh and chemically unfixed condition."	"Human temporal dura mater is mechanically highly variable with regards to its elastic modulus of 70 ± 44 MPa, tensile strength of 7 ± 4 MPa, and maximum strain of 11 ± 3 percent. Mechanical properties of the dura mater did not vary significantly between side nor sex and decreased with the age of the cadaver. Both elastic modulus and tensile strength appear to have constant mechanical parameters within the first 139 hours post mortem."

DOI = Digital object identifier, N/a = Information not available

[1]Location refers primarily to the country where the study was conducted, and secondarily to the authors' main affiliation.

[2]Primary theme refers to the main research theme that was focused on. Themes were categorized as follows: Anatomy, Asphyxia and hypoxia, Brain edema, Brain tissue identification, Drowning, Hypothermia and hyperthermia, Laboratory methods and quality, Neurodegeneration, Postmortem interval, Substance abuse, Sudden unexpected death in epilepsy, Sudden unexpected death in infancy and childhood, Suicide, Traumatic intracranial injury, Several primary entities, Other.

[3]Primary method applied in the study. Methods were categorized as follows: Conventional measurements (e.g. weight), Genetic techniques, Histology and conventional staining, Immunotechniques, Macroscopic observation, Several primary methods, Other.

[4]Total sample size, comprising both cases and controls, if applicable.

[5]Minimum and maximum ages of the total sample, comprising both cases and controls, if applicable.

[6]Aims and Main findings/conclusions mainly quote the original articles. However, the original sentences may have been truncated and essential details may have been added [using square brackets] to better convey the messages in the neuropathological context of the present review. Where possible, abbreviated terms have been written out in full.
